# Supplementary material for: A Monte Carlo analysis of false inference in spatial conflict event studies
Source: PLoS One. 2022 Apr 5;17(4):e0266010. doi: 10.1371/journal.pone.0266010 (PMC8982878; doi:10.1371/journal.pone.0266010)
Supplement: S1 File — (ZIP) [file pone.0266010.s001.zip › si.pdf]

A Monte Carlo Analysis of False Inference in Spatial Conflict Event Studies
Supplementary Information

Sebastian Schutte and Claire Kelling

Contents

1 Substantive review of published SEA designs **1**

2 Simulator resolution **3**

3 Ceiling effects in simulated locations **3**

4 Model comparison for the regression analysis **3**

5 Effects of diffusion and spatial confounders in Monte Carlo simulations **4**

6 Replication of computational experiments **5**

7 Description of the Simple Conflict Event Generator **5**

8 Description of the R-code for analysis **6**

9 Robustness tests **7**

9.1 Original results with alternative random seed 9

9.2 Higher population effect 11

9.3 Reduced diffusion range, increased error correlation 13

10 Maps of conflict countries and spatial units **14**

1 Substantive review of published SEA designs

The SEA designs summarized in Table 1 in the main manuscript have informed prominent debates. Early country-year studies have established a strong correlation between countries' overall population size and the risk of civil war (see Hegre and Sambanis 2006), yet the underlying mechanism

remained disputed. Raleigh and Hegre (2009) revisited the question with a spatial event aggregation design and found that high local population concentrations are associated with higher conflict activity.

Similarly, the link between poverty and civil war onset had been studied in numerous country-year regression designs. Hegre et al. (2009) contributed a case study for Liberia relying on events and artificial spatial units. While their study was focused on one country, Buhaug et al. (2011) took full advantage of the large geographic coverage of event data collections.

By relying on an early version of PRIO-GRID, the authors produced insights at much higher levels of resolution and found that areas of absolute poverty are indeed more prone to conflict.

Beyond causes of initial clashes, research on conflict escalation has benefited from SEA designs. Condra and Shapiro (2012) study the effects of civilian killings inflicted by either insurgents or US forces in Iraq following the 2003 invasion. Drawing on SIGACT data coded by the US military in the field and level-2 administrative units, they find that insurgent attacks on civilians do not increase subsequent insurgent activity as much as killings by US forces – presumably due to backlashes in public sentiments after US attacks. Fjelde and Hultman (2014) use PRIO-GRID to show that targeted killings of civilians in African civil wars occur more frequently in settlement areas of groups supportive of military adversaries. Pierskalla and Hollenbach (2013) also rely on PRIO-GRID to study the effects of cell-phone coverage on political violence in Africa and find that areas with cell-phone coverage see increased levels of political violence. The authors conclude that this effect results from mobile technology easing collective action problems. Manacorda and Tesei (2020) find that the effect of mobile communication technology is only salient in times of economic downturns, using data from both PRIO-GRID and GADM. Nemeth et al. (2014) apply a SEA design based on PRIO-GRID and the Global Terrorism Databse (GTD) to study determinants of hot spots of terrorist activity and corroborate central findings from the civil war literature. The effects of aid on transnational terrorism are in the focus of Boutton and Pascoe (2018). The authors report increases in the probability of terrorist attacks in PRIO-GRID cells that overlap with aid projects. Elliott and Kreutz (2019) find that anticipations of short remaining conflict duration increase fighting over natural resources and base their analysis on PRIO-GRID data.

Reductions in violence have also been researched based on SEA designs. Wood and Sullivan (2015) research the effects of war-time aid provision on subsequent conflict dynamics and find that aid can have a conflict-inducing effect due to increased opportunities for looting and challenged rebel control over resources. Duursma (2019) researches obstacles to effective peace keeping and rely on PRIO-GRID in a supplementary analysis. Fjelde et al. (2019) find that peacekeeping is an effective tool to reduce levels of violence. Finally, SEA designs have also been used to assess the climate-conflict link. While conventional wisdom suggests that global warming could directly contribute to increased risks of civil war, Theisen et al. (2012) showed that evidence of local weather anomalies leading to full-blown civil wars is scarce. Utilizing data on administrative units, Fjelde and von Uexkull (2012) added that reduced local precipitation can increase the risk of communal violence instead of civil war in Sub-Saharan Africa. Ide et al. (2014) rely on various spatial data collections to identify regions of particular risk for climate-change related conflict. O'Loughlin et al. (2014) research the climate-conflict link in Africa and find that different regions vary widely in their resilience to drought and extreme temperatures. The authors merge data of various spatial resolutions and conduct analyses across aggregations.

2 Simulator resolution

Internally, the simulator represents the bounding box of each country in a finite lattice of 600x400 discrete locations. This entails that these discrete locations account for different areas in the real world, depending on country size and layout. The simulated cell sizes in meters are as follows: Afghanistan: 2,621x2,620; Colombia: 4,714x4,710; Pakistan: 3,799x3,809; Iraq: 2,369x2,369; Somalia: 3,810x3,820; Turkey: 2,897x2,893; Nepal: 1,373x1,372. The discrepancies between latitudinal and longitudinal extent are very small thanks to local UTM projections. For each computational experiment, the smallest geographic aggregation (PG0.025) corresponds to 27,750x27,750 meters at the equator.

3 Ceiling effects in simulated locations

We simulate conflict events in small simulated locations as a series of Bernoulli trials: in several steps (ten in our case), simulated locations get a chance to 'produce' a conflict event. If parameters were chosen incorrectly, this could lead to censoring. If the simulation parameters are chosen such that even small population numbers generate a conflict event with near certainty, then locations with higher population numbers will also only produce a single conflict event. We mitigate this problem in two ways: first, we choose parameters generally to re-create empirically observed numbers of events. To do this, we factor in the total expected number of events, total population counts, number of simulated locations, and the number of simulated time steps.

We therefore have no use case where astronomically high event numbers need to be simulated. Second, we generate a series of events over the course of several steps (10 for the current batch of results). The number of trials was chosen to minimize the number of simulated locations producing 10 events in 10 trials, given the event probabilities necessary to recreate the empirically observed N.

We have double-checked that our parameter choices work. In Figure A2 on page 17, we show that we do not encounter ceiling effects due to censoring at the level of simulated locations for the simulated countries (first run, experiment 1a).

4 Model comparison for the regression analysis

We chose linear models to regress event counts on population numbers at the level of PRIO-GRID and admin units. As event counts and variance in the cross-sectional analysis are generally high, we presumed linear models would result in better fits than count-dependent variables models, such as Poisson models. We validate this assumption below in Figure A1 on page 16. We find that linear models usually result in lower AIC scores (i.e. better fit) than Poisson models. For this comparison, we re-estimated both model types as GLMs for experiments 1a and 3a, both of which feature a true population effect.

5 Effects of diffusion and spatial confounders in Monte Carlo simulations

We study problems and remedies to inference in SEA designs. One prerequisite for this is that the simulated mechanisms lead to the expected statistical problems. In this section, we show that the presented diffusion mechanism leads to spatial autocorrelation which can be identified with Moran tests. Also, we show that the error correlation scenario leads to higher rates of non-normality in the distribution of the residuals.

We conduct two sets of statistical tests identified in the literature. Ward and Gleditsch (2008:33) point to the Moran's I statistic to identify spatial autocorrelation between neighboring units for all event datasets. In a first set of tests, we run Moran tests for spatial autocorrelation between neighboring units. The results are displayed in Figure A3 on the left. We compare experiments 1a and 2a, i.e. situations where a true population effect is present and diffusion is absent and present respectively.

Diffusion of violence leads to systematically increased rates of passed Moran tests (as seen in higher rates of significant results for the Moran tests in the lower rows for each country). This effect is especially pronounced for the PRIO-GRID cells, but also clearly discernible for ADM1 aggregations. However, Moran tests for ADM2 aggregations also pass in the absence of diffusion effects. This can be due to the spatial clustering of population at these aggregations which leads to clustered events in the absence of diffusion. We conclude that Moran tests are an eligible tool for the detection of diffusion processes and that Spatial lag models effectively solve the intended problem. Alternative diffusion mechanisms could be simulated, but there is little published research into which exact mechanisms apply across cases.[1]

For the analysis of spatial error correlation, we rely on a different statistical test, but the same overall approach. We attempt to generate this effect in the simulations by introducing military bases that randomly increase or decrease conflict activity in their vicinity. Both in experiment 1a (population effect) and 3a (population effect and military bases), we estimate linear models with area controls. According to Ward and Gleditsch (2008:33), non-normality in the distribution of residuals can be indicative of unmodeled spatial confounders that are themselves spatially correlated.

We therefore analyze the residuals in linear models in the presence and absence of bases by conducting Shapiro-Wilk tests on the residuals. Higher counts indicate higher rejection rates for normally distributed errors in Figure A3 (right side). For both PRIO-GRID aggregations, non-normality in the residuals is almost universal. Linear models based on experiment 3a show slightly higher counts of significant results. A similar picture emerges for the ADM1 level, but differences between the experiments are small on average.

These results are subject to parameter choices. In section 9, we repeat this analysis with lower diffusion ranges and larger areas subject to correlated errors. As expected, the test statistics show higher discrepancies for Shapiro-Wilk tests and lower discrepancies for the Moran tests.

One striking results remains, however: differences across units and countries dominate differences in the DGP. We conclude that the problems introduced by Modifiable Areal Units dominate those caused by our implementation of spatial diffusion and unmodeled confounders. However, our implementation of problems in the simulator leads to the predicted statistical effects and previously suggested diagnostic tests can work as intended (Ward and Gleditsch 2008).

[1](Zhukov 2012) suggests that violence should relocate along road networks and this could be researched in the future, but it lies beyond the scope of this paper.

6 Replication of computational experiments

To replicate the computational experiments, the Simple Conflict Event Generator (SCEG) and R code must be downloaded from `https://github.com/prio-data/climsec_plos22_replication/`. Replication data must be obtained from `https://dataverse.harvard.edu/dataset.xhtml?persistentId=doi:10.7910/DVN/FBR16C`.

Once data and code are downloaded place copy the "inputs" folder from dataverse into the "R_code" folder obtained from github. Copy the "country_data" folder from dataverse into the "netlogo_code" folder obtained from github. This will make the simulator and the analysis code executable. SCEG requires the NetLogo runtime and programming environment to be installed (version 6.11, see `https://ccl.northwestern.edu/netlogo/download.shtml`). Once loaded within NetLogo, SCEG can be used to generate conflict event data for every country that was in existence in 2010. For replication purposes, the experiments presented in the main article are pre-configured including random seeds that ensure consistent results across repetitions. The seven countries, Afghanistan, Colombia, Pakistan, Iraq, Somalia, Turkey, and Nepal can be selected in the drop-down menu and the "Generate Data!" button will generate 100 event datasets in one csv file per country. Please specify correct paths in the graphical user interface in advance. Several Netlogo instances can be opened simultaneously to take advantage of multi-core CPUs, but event simulation still requires several hours to complete. The total disk space required for event data is approximately 2.1GB. Note that the R analysis scripts require the installation of several libraries within R.

7 Description of the Simple Conflict Event Generator

SCEG is written in the NetLogo scripting language. The graphical user interface (shown in Figure A4) can be used to generate artificial event datasets. To this end, a country of interest from the CShapes list be chosen, the DGP has to be configured, and an output file for event datasets has to be named. For creation of batches of conflict events, sets of parameters can be added to the simulator code.

SCEG performs the following functions:

- Wait for the user to select a country in the graphical interface.
- Upon a press of the *setup* button, the simulator space is initialized. To this end, country boundaries from the CShapes data collection are loaded, corresponding to the 2010 situation (Weidmann et al. 2010). Population data for the countries is loaded from the WorldPop dataset (Worldpop 2018). Geographic cell sizes for the simulated locations are calculated and the population data is resampled to match the simulator resolution, to prevent double counting. All simulated locations within the relevant country are associated with population and their internal violence counters are set to zero. Also, geographic coordinates of each cell are established. For performance reasons, each cell is associated with a lookup table for random values for each simulated time step. Bases are randomly distributed in the country polygon and each simulated location is associated with the nearest base. The bases are associated with a marginal causal effect on conflict probability in their vicinity.
- After the setup is complete, the *go* button can be pressed for single runs or the *generate data* button for batch runs. The go loop runs through the following steps:

  - All simulated locations calculate the probability of witnessing an attack based on the chosen DGP. This calculation involves their population levels, possibly the the effect of the nearest base, and the overall baseline probability.
  - Each location looks up a random real number between 0 and 1 in the pre-calculated lookup table.
  - If the calculated attack probability is larger than the drawn random number, an attack is recorded.
  - If diffusion of violence is to be simulated, a location in the vicinity is chosen at random to record the attack, within the diffusion range.

- After a configured number of simulation steps, the run concludes and the resulting event dataset including geographic coordinates is written to the output file.

8 Description of the R-code for analysis

The file *01_plot_shapefiles.R* contains replication code for the figures of all of the shapefiles included in Section 10 of the Suppplementary Information. The file *02_replication_code_many_countries.R* replicates our analysis for all countries considered in the manuscript. The code sets the user's working directory to the location of the file. The code lists the cases to consider (all cases considered in this manuscript are included) and draws from the input/ folder in the directory for shapefiles and event data.

The code performs the following steps:

- Load all helper functions from *00_functions_repcode.R* for use in the analysis.
- Cycle through every case (combination of country, event data, areal units, and regression equation).
- The function *full_function* takes the country of interest, event data, and the name of the units as arguments (all provided by the case considered). It performs the following tasks:
  - Load the unit-specific shapefile of the studied country from the input folder. For instance, this could be Afghanistan at the ADM2 level. All units have been coded with population totals based on WorldPop.
  - Load the event data, which has been generated by SCEG.
  - For each experiment and specific simulation run (specified with a run_id in the event data), superimpose the events on the shapefile.
  - Return a dataframe that includes the number of events and the population per unit along with the run_id and experiment number.
  - Return a list of the following format:
    * The first item in the list is a list of the dataframes. Each dataframe in the list represents one run_id and one experiment.
    * The second item is the shapefile of interest.

- For each experiment and unit, subsequent statistical analysis is performed: Take the shape file of interest returned by *full_function* and subset it to only the units that are included in the analysis.

- Create a neighborhood matrix for use in spatial modeling.

- Then we specify the model type. It could be a linear model with no spatial error structure ("lm"), a spatial lag model ("spatial_lag"), or a spatial error model ("spatial_error"). We have included examples of all three model types.

- Run the "eval_exp" function to run the model specified in the previous step.
  - For each dataframe in the list returned by full_function (for every combination of experiment and run_id), run the model specified by the model type and model form, given by the user.
  - Return the sign (positive or negative) and significance of each coefficient specified by the model form.
  - Next, iterate through the experiments. For each experiment, aggregate all runs to find the percentage of runs that had positive and significant coefficients, negative and significant coefficients, and no effect. Perform this analysis for each coefficient and also record the model and experiment number.
  - This function returns a list where each item corresponds to a coefficient. Therefore, if there are three coefficients in the model, the list will be of length three.
  - This function also conducts Moran's I and Shapiro tests and compares the AIC from a linear model to GLM.

- We then write this dataframe to the working directory with the name of units and the model type in the name of the file. We also write the results for Moran's and Shapiro tests and the AIC comparison.

- The dataframes are then used by the plotting code in *03_plotting_results.R* to recreate the figures of the manuscript. The file *00_plot_functions.R* provides helper functions for loading data.

Several other scripts were used to code the exact coefficients for the SCEG simulator and to establish population totals for countries and units. We will share these scripts upon request.

9 Robustness tests

In this section, we present versions of the results in the main text based on alternative parameter choices. The simulation results are robust to these changes in substantive terms. First, we replicate the main analysis with a different random number sequence in SCEG (random seed "123" instead of "12345"). This does not change the results noticeably. Then, we implement a higher population effect, which generally leads to better inference. Finally, we reduce the diffusion range and increase the number of simulated bases that introduce spatial error correlation. The diagnostic statistics for the error correlation perform better in this last test, but spatial lag model still outperform the spatial error term models in the benchmarks. Table A1 summarizes the robustness tests.

Main analysis				
Exp.	Effect	Diffusion	PG 0.5 cells per base	Problems
1a	Yes (50% N)	0 km	0	MAUP and areal confounding
1b	No	0 km	0	MAUP and areal confounding
2a	Yes (50% N)	100km	0	Spatial autocorrelation
2b	No	100km	0	Spatial autocorrelation
3a	Yes (50% N)	0 km	9	Error correlation
3b	No	0 km	9	Error correlation
4a	Yes (50% N)	100km	9	All of the above
4b	No	100km	9	All of the above
Higher population effect				
Exp.	Effect	Diffusion	PG 0.5 cells per base	Problems
1a	Yes (80% N)	0 km	0	MAUP and areal confounding
1b	No	0 km	0	MAUP and areal confounding
2a	Yes (80% N)	100km	0	Spatial autocorrelation
2b	No	100km	0	Spatial autocorrelation
3a	Yes (80% N)	0 km	9	Error correlation
3b	No	0 km	9	Error correlation
4a	Yes (80% N)	100km	9	All of the above
4b	No	100km	9	All of the above
Higher ranges for diffusion and error correlation				
Exp.	Effect	Diffusion	PG 0.5 cells per base	Problems
1a	Yes (50% N)	0 km	0	MAUP and areal confounding
1b	No	0 km	0	MAUP and areal confounding
2a	Yes (50% N)	50km	0	Spatial autocorrelation
2b	No	50km	0	Spatial autocorrelation
3a	Yes (50% N)	0 km	18	Error correlation
3b	No	0 km	18	Error correlation
4a	Yes (50% N)	50km	18	All of the above
4b	No	50km	18	All of the above

Table A1: Overview of the eight data-generating processes run for all countries in the main analysis. In each experiment, 100 datasets were generated. Beyond the main results, we have repeated the whole analysis three times to test the effects of an alternative random seed for the pseudo-random number sequence in SCEG, higher population effects, and higher spatial ranges for diffusion and error correlation.

9.1 Original results with alternative random seed

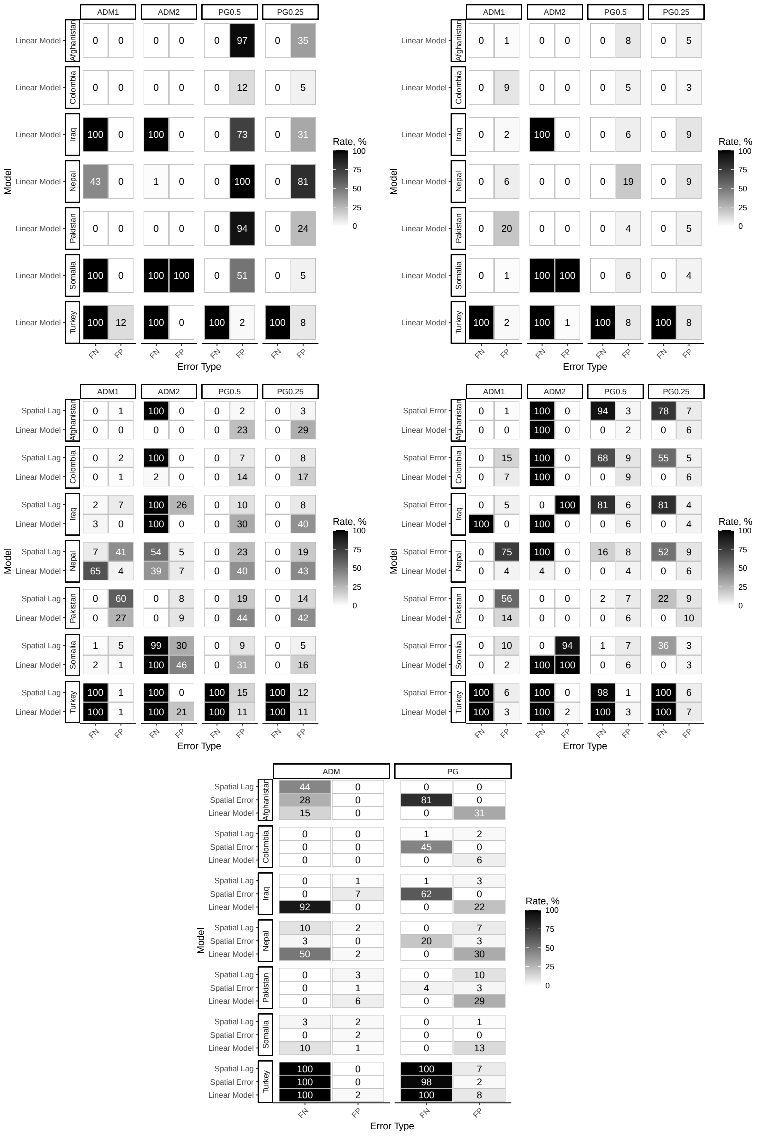

Figure A5: Main results replicated with alternative random seed (“123” instead of “12345”). The results are identical.

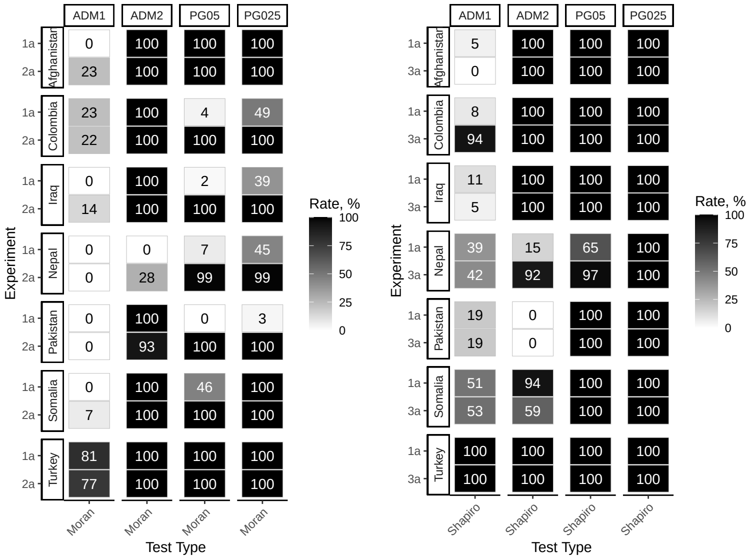

Figure A6: Rates of significant results for Moran tests for experiments 1a and 2a (left) and Shapiro-Wilk test for experiments 1a and 3a (right).

9.2 Higher population effect

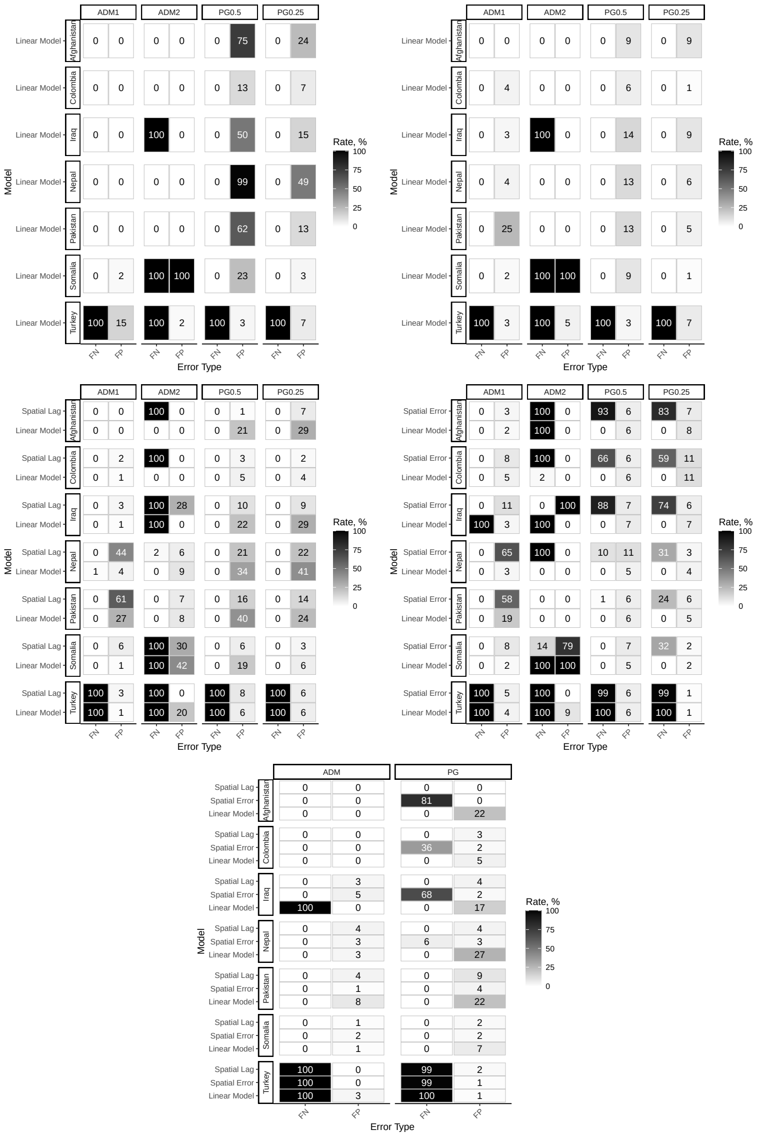

Figure A7: Main results replicated with a higher population effect. In this scenario, population accounts for 80% of conflict events. Instances of false inference are generally reduced due to the more favorable signal-to-noise ratio.

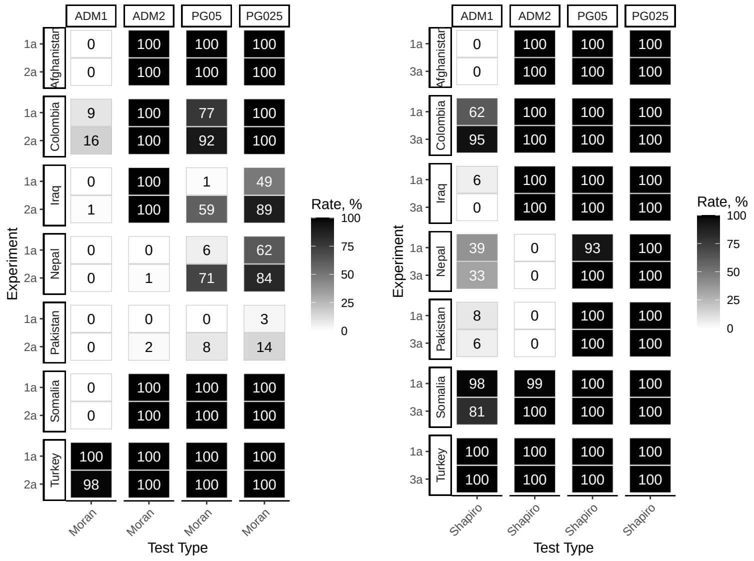

Figure A8: Rates of significant results for Moran tests for experiments 1a and 2a (left) and Shapiro-Wilk test for experiments 1a and 3a (right).

9.3 Reduced diffusion range, increased error correlation

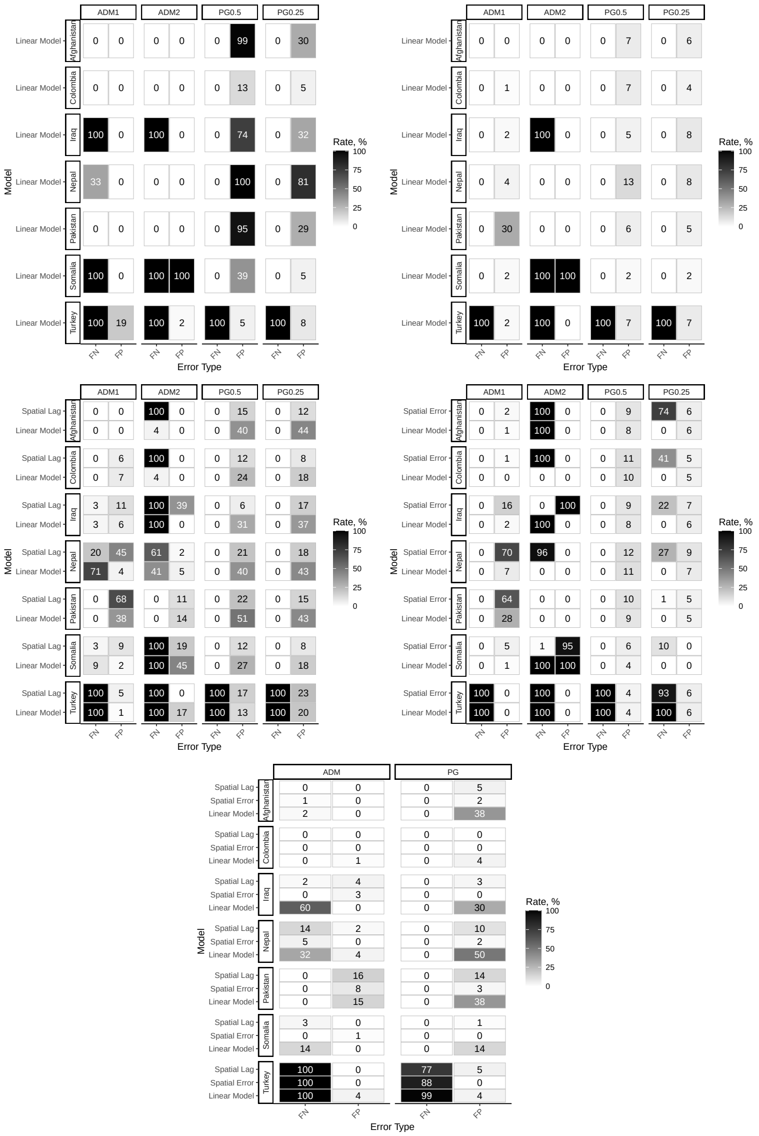

Figure A9: Main results replicated with reduced diffusion range, but larger areas of spatially correlated errors. In this scenario, the spatial error models outperform the spatial lag models in the final benchmarks.

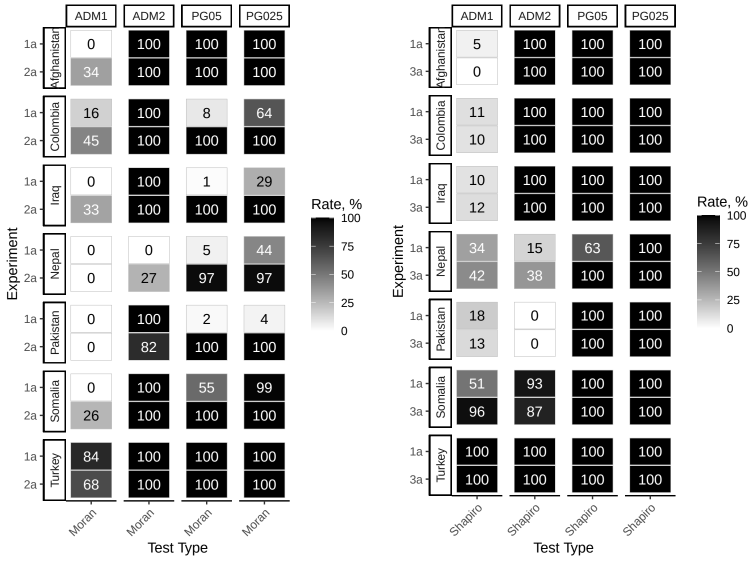

Figure A10: Rates of significant results for Moran tests for experiments 1a and 2a (left) and Shapiro-Wilk test for experiments 1a and 3a (right). The Shapiro-Wilk tests for non-normality in the residuals succeed at higher rates in this scenario for the bottom rows. This suggests that the test is eligible in situations of more severe error correlation.

10 Maps of conflict countries and spatial units

References

Andrew Boutton and Henry Pascoe. Do foreign aid projects attract transnational terrorism? *Peace Economics, Peace Science and Public Policy*, 24(4), 2018.

Halvard Buhaug, Kristian Skrede Gleditsch, Helge Holtermann, Gudrun Østby, and Andreas Forø Tollefsen. It's the local economy, stupid! geographic wealth dispersion and conflict outbreak location. *Journal of Conflict Resolution*, 55(5):814–840, 2011.

Luke N Condra and Jacob N Shapiro. Who takes the blame? the strategic effects of collateral damage. *American Journal of Political Science*, 56(1):167–187, 2012.

Allard Duursma. Obstruction and intimidation of peacekeepers: How armed actors undermine civilian protection efforts. *Journal of Peace Research*, 56(2):234–248, 2019.

Kaisa Hinkkainen Elliott and Joakim Kreutz. Natural resource wars in the shadow of the future: Explaining spatial dynamics of violence during civil war. *Journal of Peace Research*, 56(4): 499–513, 2019.

Hanne Fjelde and Lisa Hultman. Weakening the enemy: A disaggregated study of violence against civilians in africa. *Journal of Conflict Resolution*, 58(7):1230–1257, 2014.

Hanne Fjelde and Nina von Uexkull. Climate triggers: Rainfall anomalies, vulnerability and communal conflict in sub-saharan africa. *Political Geography*, 31(7):444–453, 2012.

Hanne Fjelde, Lisa Hultman, and Desirée Nilsson. Protection through presence: Un peacekeeping and the costs of targeting civilians. *International Organization*, 73(1):103–131, 2019.

Håvard Hegre and Nicholas Sambanis. Sensitivity analysis of empirical results on civil war onset. *Journal of conflict resolution*, 50(4):508–535, 2006.

Håvard Hegre, Gudrun Østby, and Clionadh Raleigh. Poverty and civil war events: A disaggregated study of liberia. *Journal of Conflict Resolution*, 53(4):598–623, 2009.

Tobias Ide, Janpeter Schilling, Jasmin SA Link, Jürgen Scheffran, Grace Ngaruiya, and Thomas Weinzierl. On exposure, vulnerability and violence: spatial distribution of risk factors for climate change and violent conflict across kenya and uganda. *Political Geography*, 43:68–81, 2014.

Marco Manacorda and Andrea Tesei. Liberation technology: mobile phones and political mobilization in africa. *Econometrica*, 88(2):533–567, 2020.

Stephen C Nemeth, Jacob A Mauslein, and Craig Stapley. The primacy of the local: Identifying terrorist hot spots using geographic information systems. *The Journal of Politics*, 76(2):304–317, 2014.

John O'Loughlin, Andrew M Linke, and Frank DW Witmer. Effects of temperature and precipitation variability on the risk of violence in sub-saharan africa, 1980–2012. *Proceedings of the National Academy of Sciences*, 111(47):16712–16717, 2014.

Jan H Pierskalla and Florian M Hollenbach. Technology and collective action: The effect of cell phone coverage on political violence in africa. *American Political Science Review*, 107(2):207–224, 2013.

Clionadh Raleigh and Håvard Hegre. Population size, concentration, and civil war. a geographically disaggregated analysis. *Political geography*, 28(4):224–238, 2009.

Ole Magnus Theisen, Helge Holtermann, and Halvard Buhaug. Climate wars? assessing the claim that drought breeds conflict. *International Security*, 36(3):79–106, 2012.

Michael D Ward and Kristian Skrede Gleditsch. *Spatial regression models*, volume 155. Sage Publications, 2008.

Nils B Weidmann, Doreen Kuse, and Kristian Skrede Gleditsch. The geography of the international system: The cshapes dataset. *International Interactions*, 36(1):86–106, 2010.

Reed M Wood and Christopher Sullivan. Doing harm by doing good? the negative externalities of humanitarian aid provision during civil conflict. *The Journal of Politics*, 77(3):736–748, 2015.

Worldpop. www.worldpop.org - school of geography and environmental science, university of southampton; department of geography and geosciences, university of louisville; departement de geographie, universite de namur and center for international earth science information network (ciesin), columbia university (2018). global high resolution population denominators project - funded by the bill and melinda gates foundation (opp1134076), 2018. URL `www.worldpop.org`.

Yuri M. Zhukov. Roads and the diffusion of insurgent violence: The logistics of conflict in russia's north caucasus. *Political Geography*, 31(3):144–156, 2012.

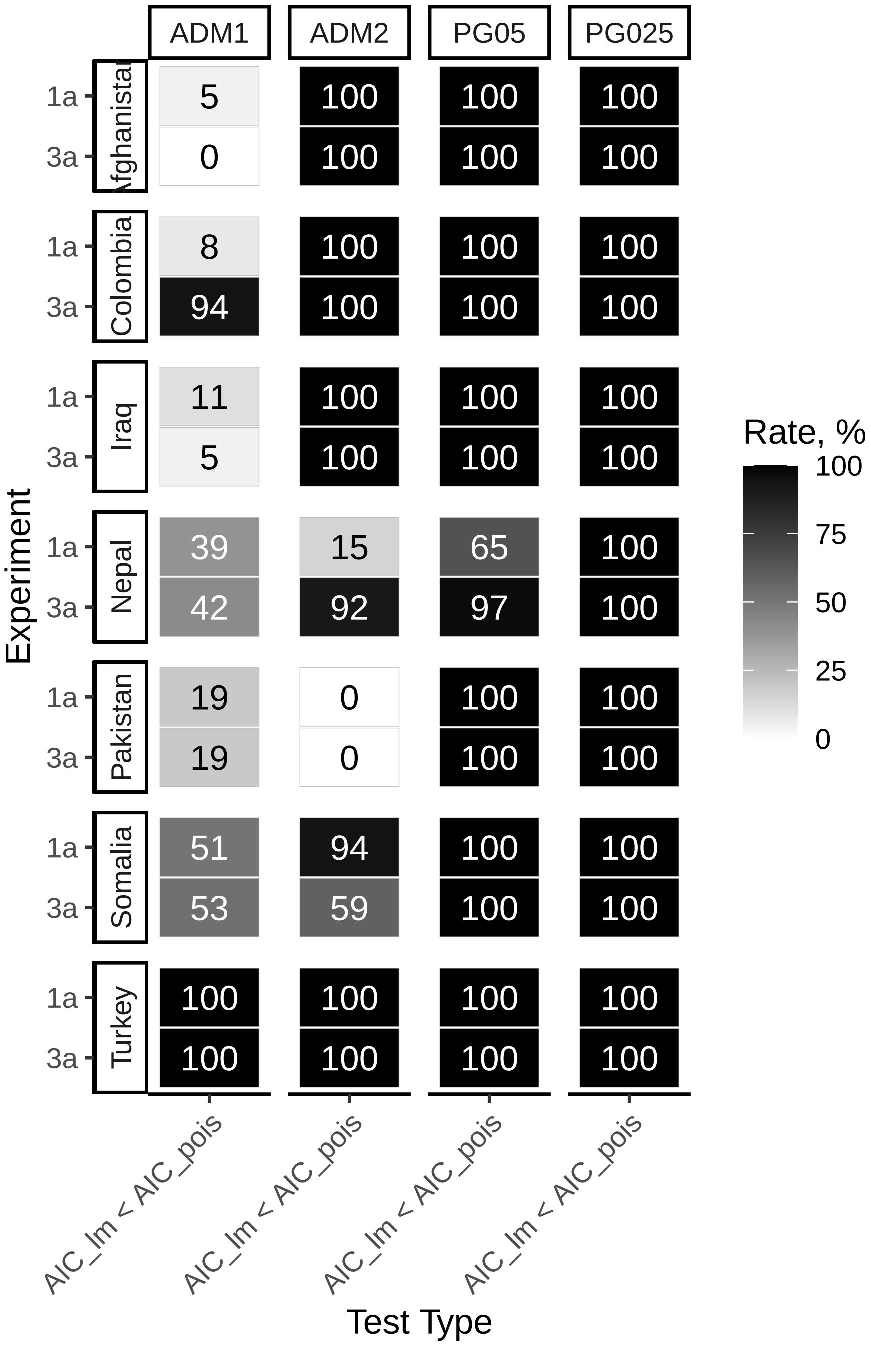

Figure A1: **Comparison between linear models and Poisson models for the regression of event counts on population numbers for PRIO-GRID and administrative units. We find that for most simulated countries and units, linear models perform better in AIC comparisons. This is likely due to the fact that event counts and variance in our cross-sectional analysis tend to be high.**

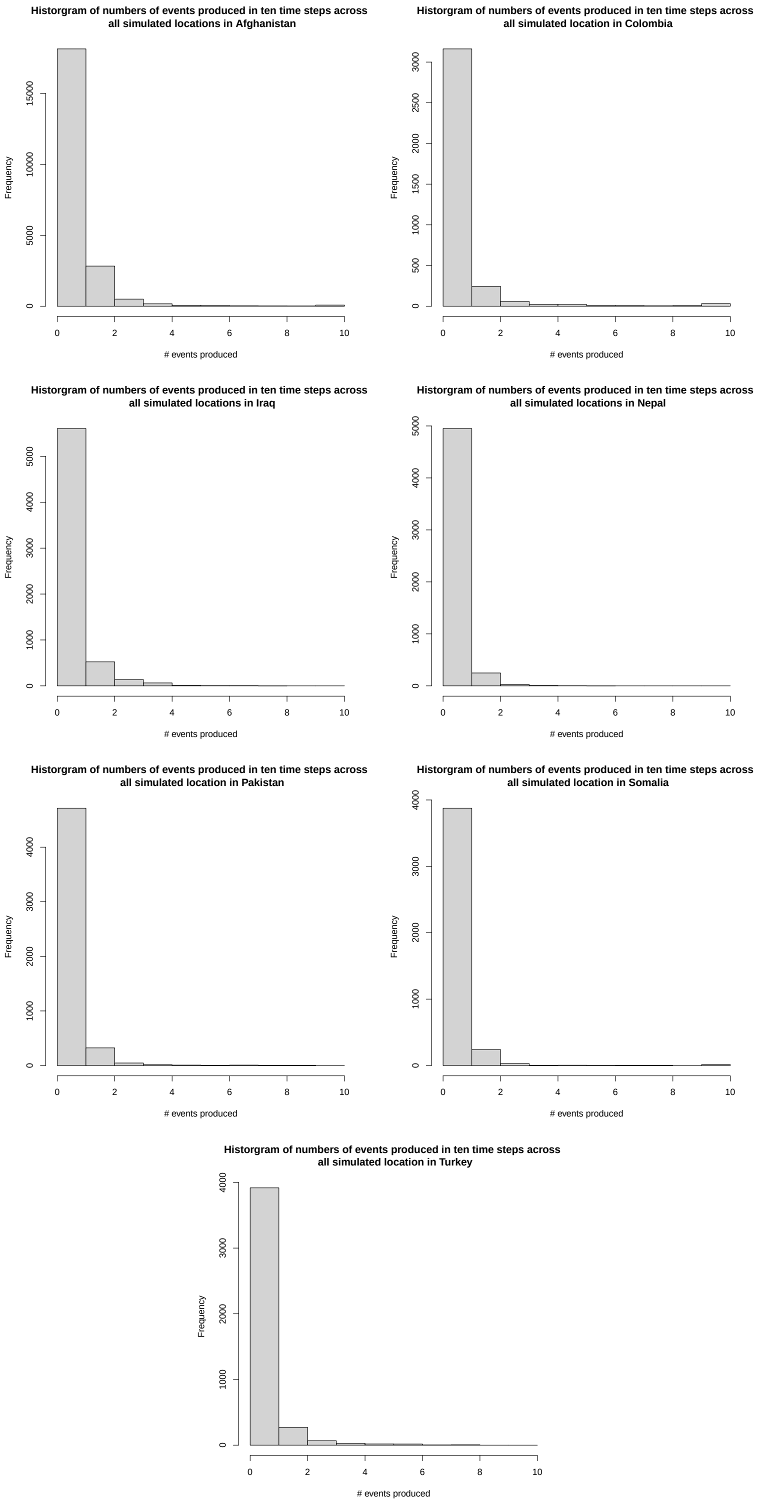

Figure A2: Absence of ceiling effects for event counts in simulated locations.

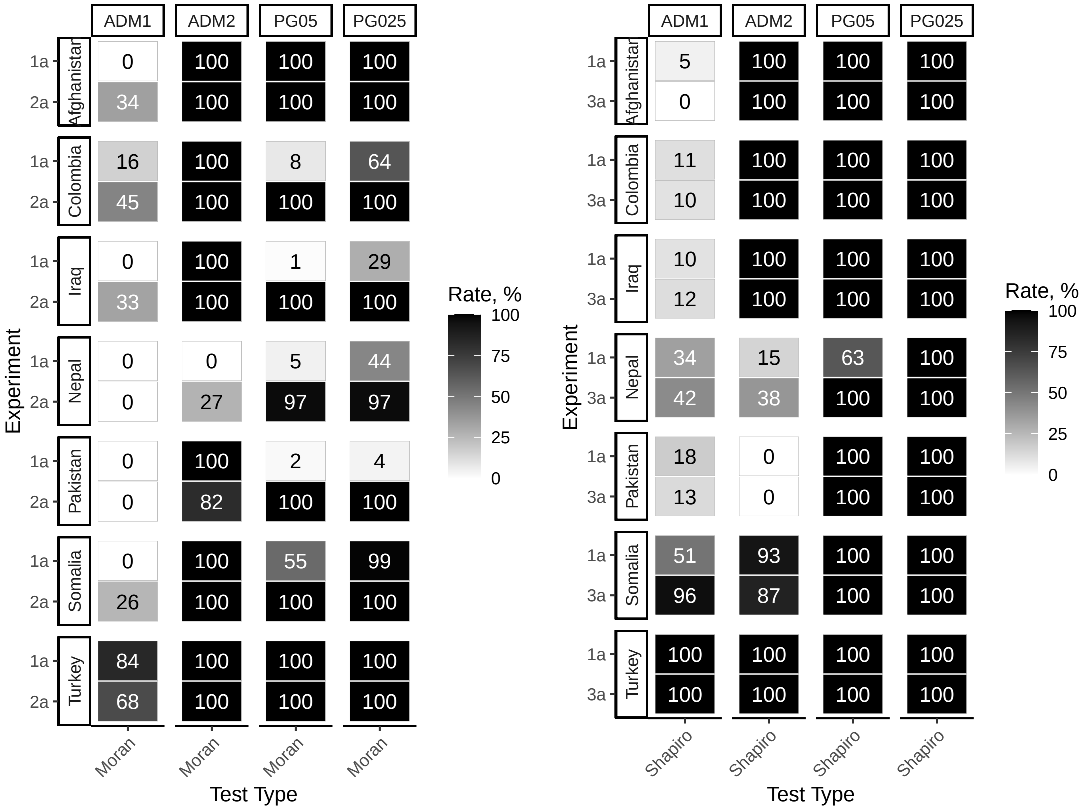

Figure A3: Rates of significant results for Moran tests (for spatial autocorrelation) for experiments 1a and 2a (left) and Shapiro-Wilk test (for non-normality in the residuals) for experiments 1a and 3a (right). Area controls are included. The rate is the percentage of runs that had a significant test statistic.

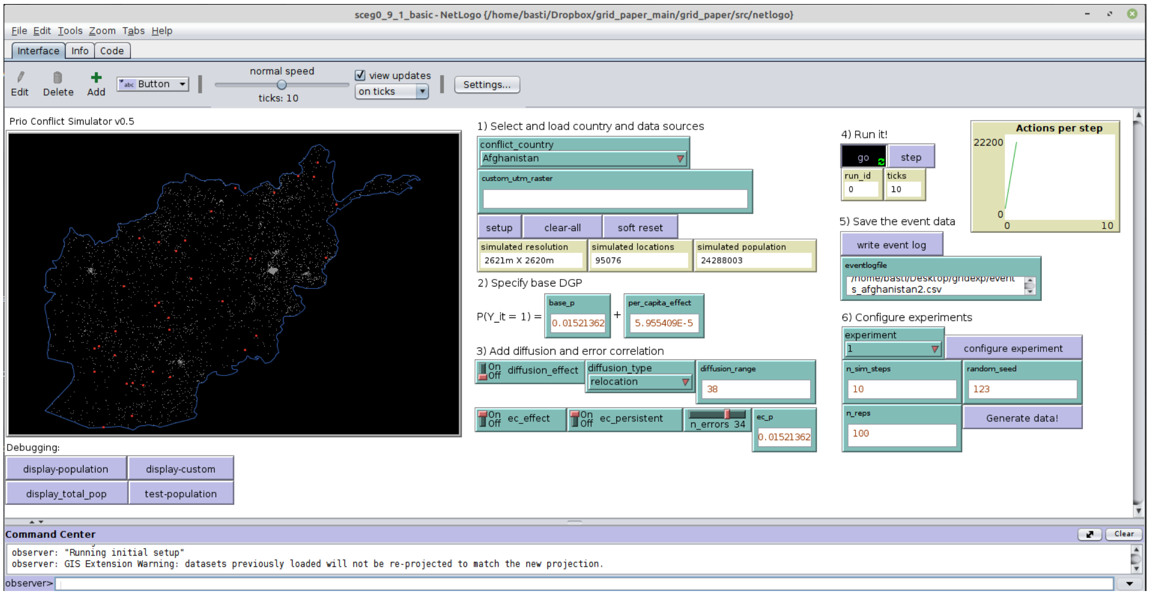

Figure A4: Screenshot of SCEG

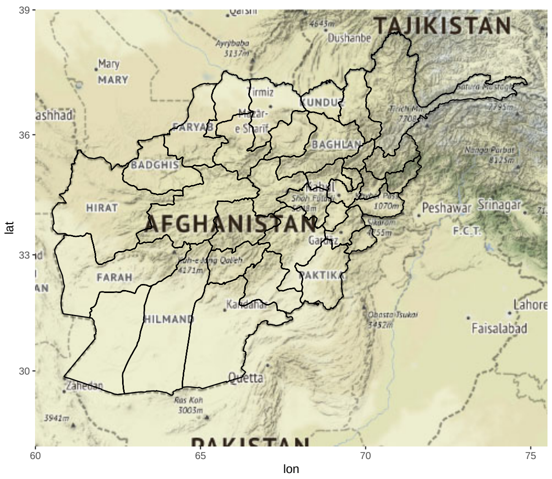

(a) ADM1

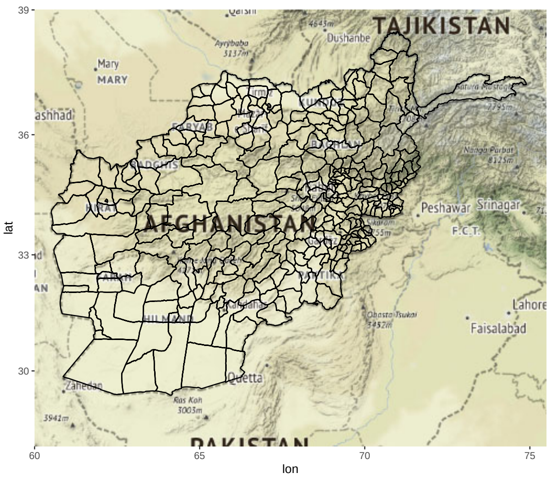

(b) ADM2

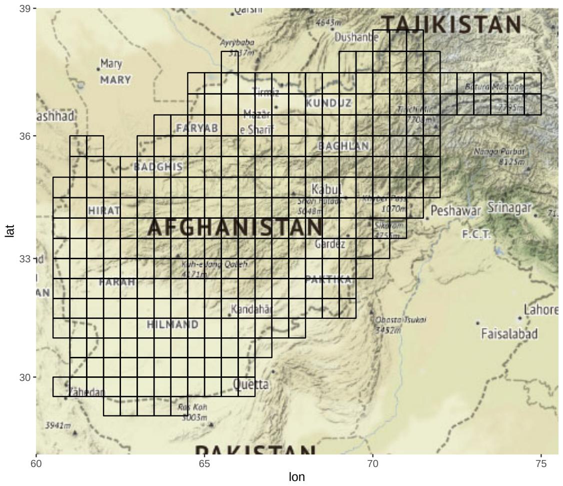

(c) PRIOGrid 0.5

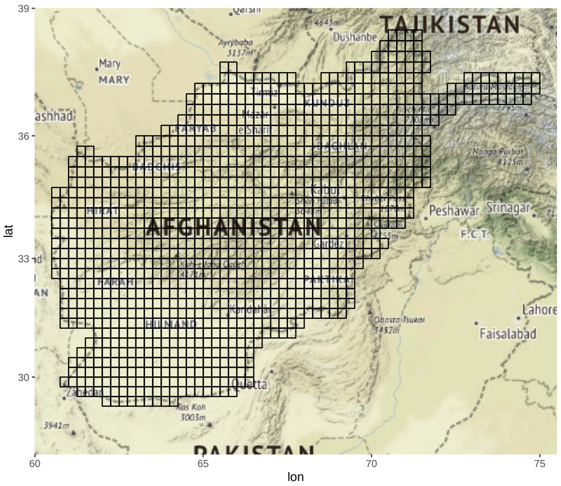

(d) PRIOGrid 0.5

Figure A11: Units of analysis in Afghanistan.

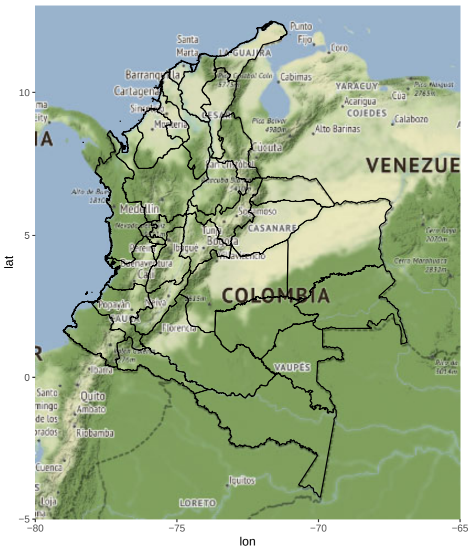

(a) ADM1

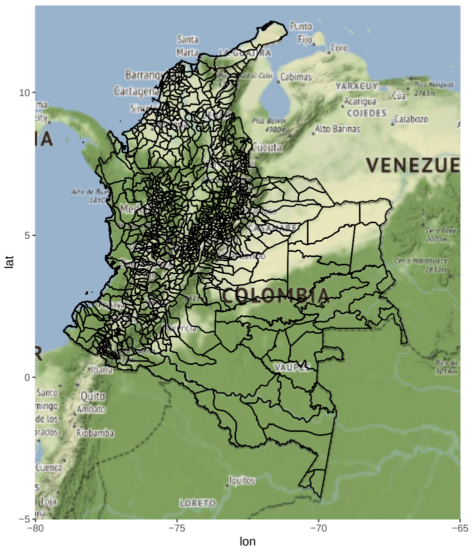

(b) ADM2

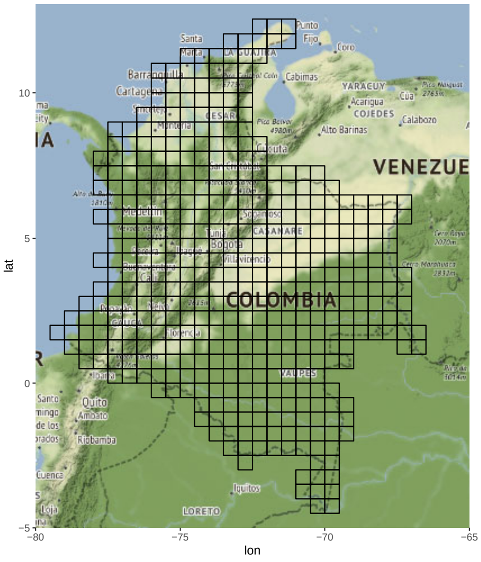

(c) PRIOGrid 0.5

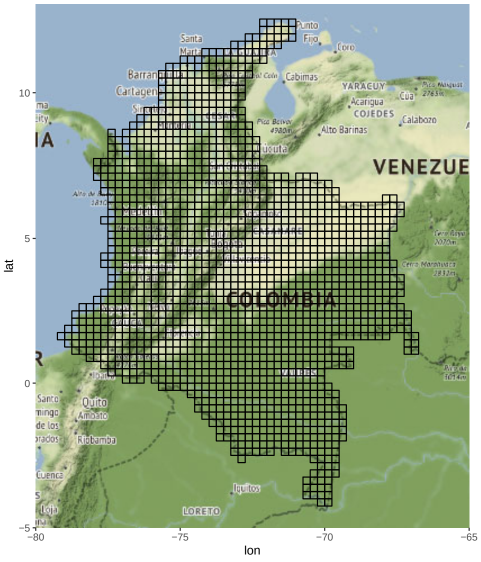

(d) PRIOGrid 0.5

Figure A12: Units of analysis in Colombia.

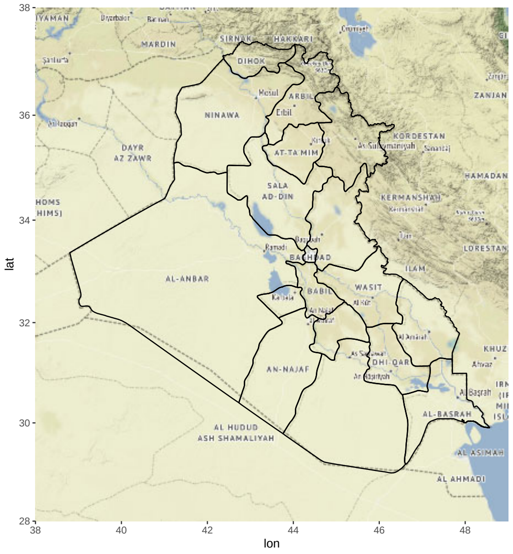

(a) ADM1

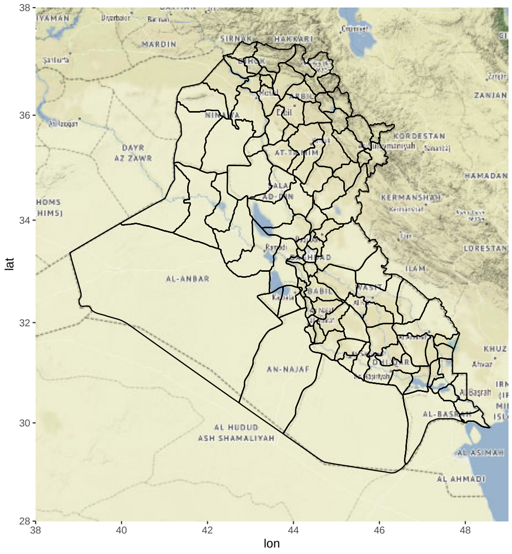

(b) ADM2

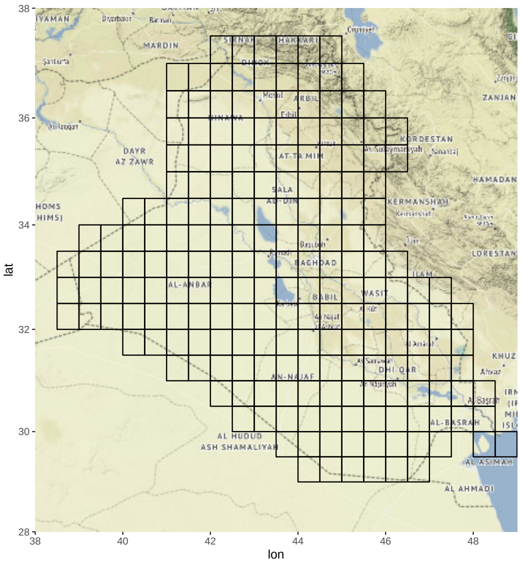

(c) PRIOGrid 0.5

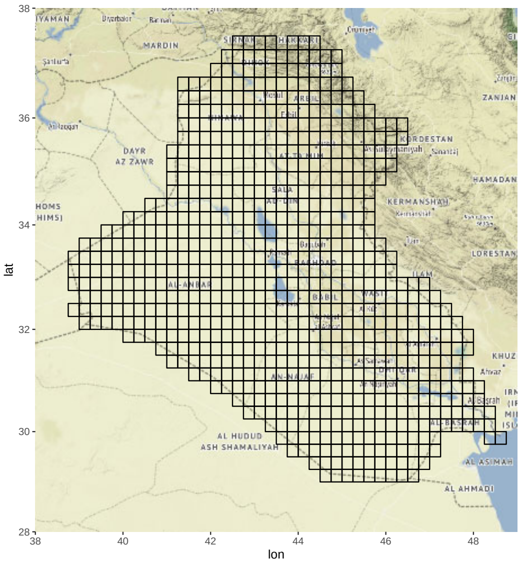

(d) PRIOGrid 0.5

Figure A13: Units of analysis in Iraq.

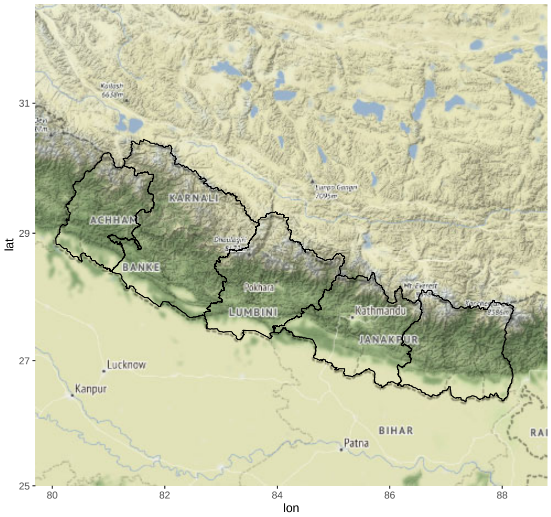

(a) ADM1

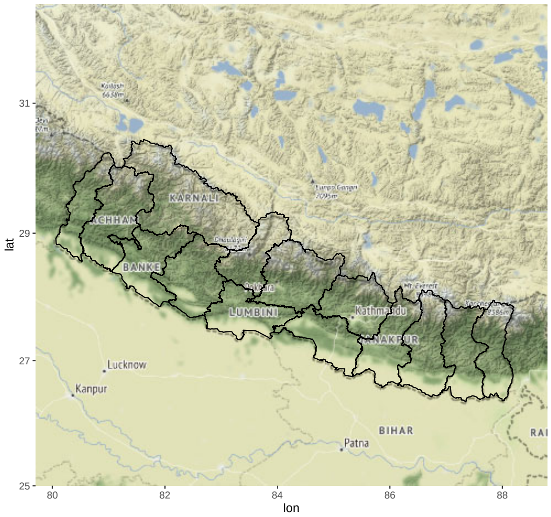

(b) ADM2

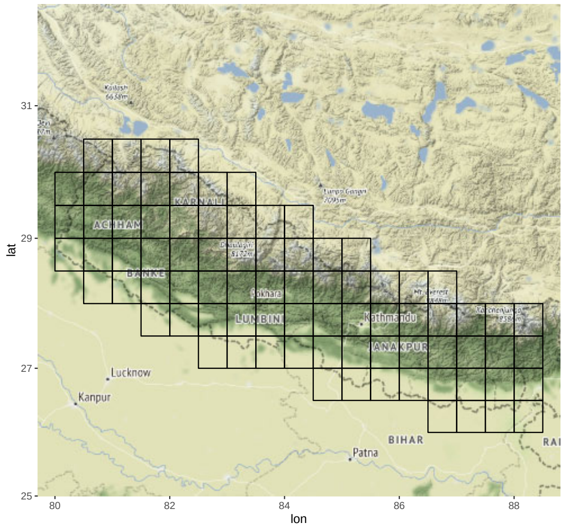

(c) PRIOGrid 0.5

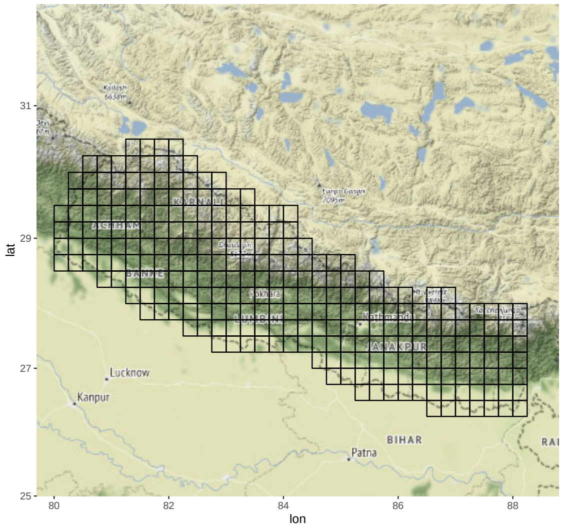

(d) PRIOGrid 0.5

Figure A14: Units of analysis in Nepal.

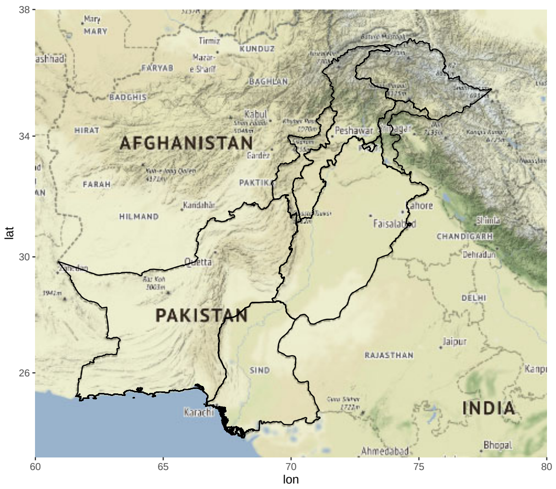

(a) ADM1

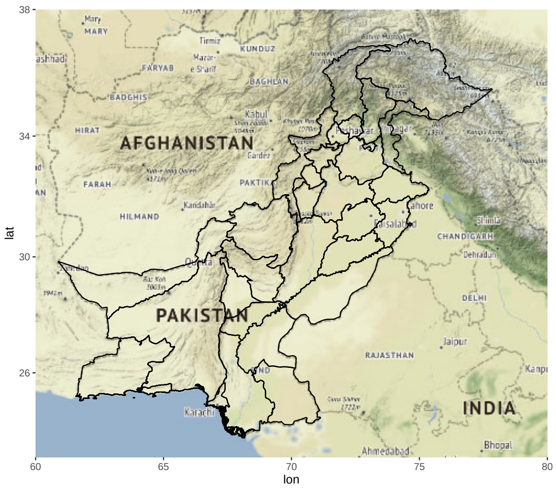

(b) ADM2

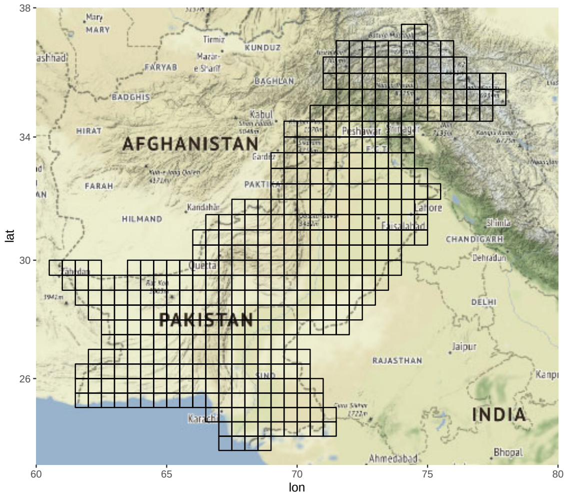

(c) PRIOGrid 0.5

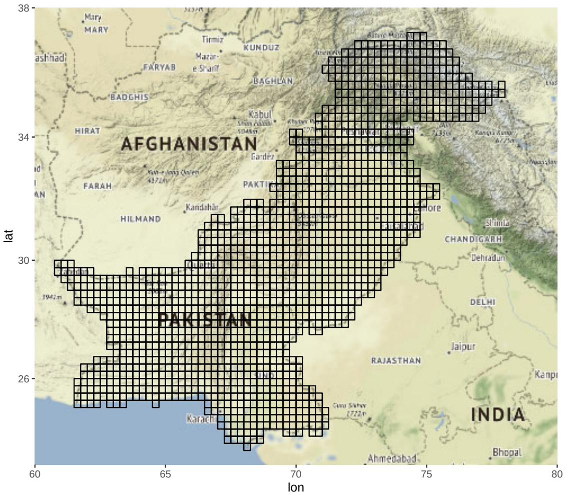

(d) PRIOGrid 0.5

Figure A15: Units of analysis in Pakistan.

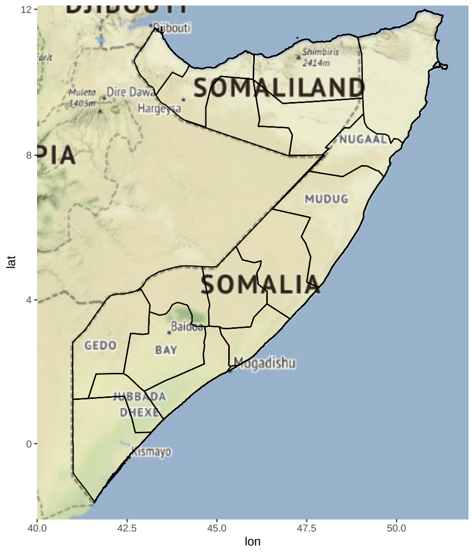

(a) ADM1

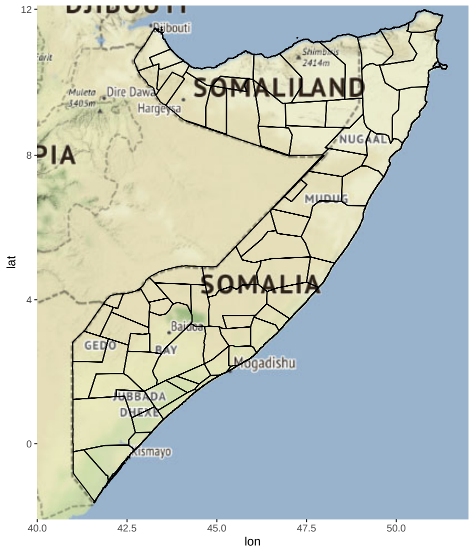

(b) ADM2

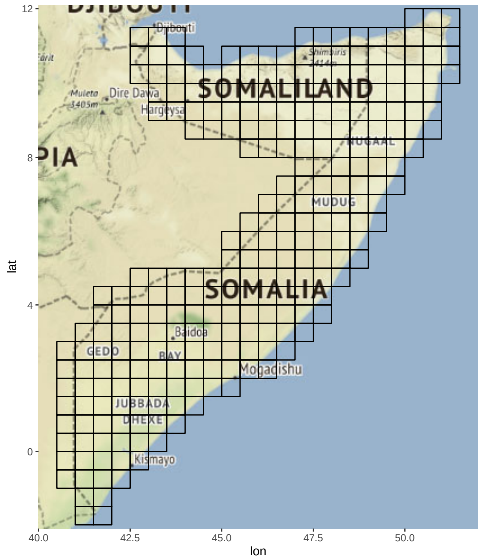

(c) PRIOGrid 0.5

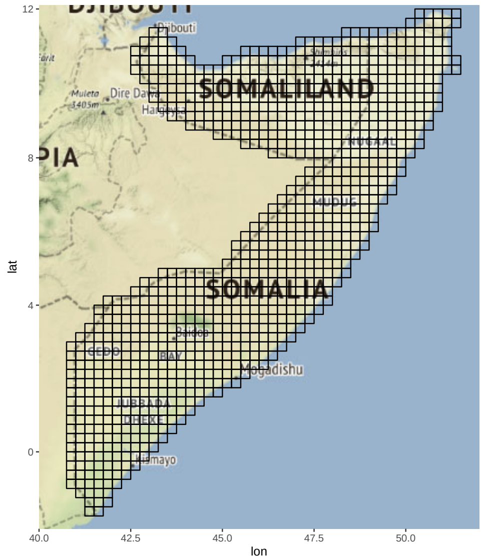

(d) PRIOGrid 0.5

Figure A16: Units of analysis in Somalia.

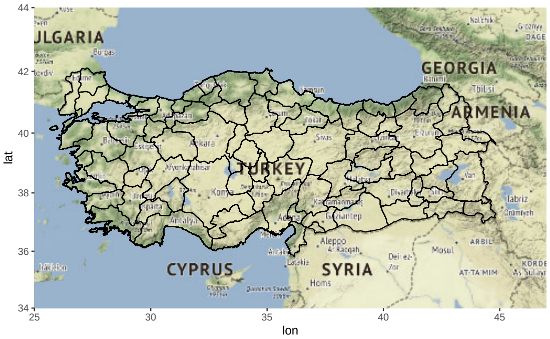

(a) ADM1

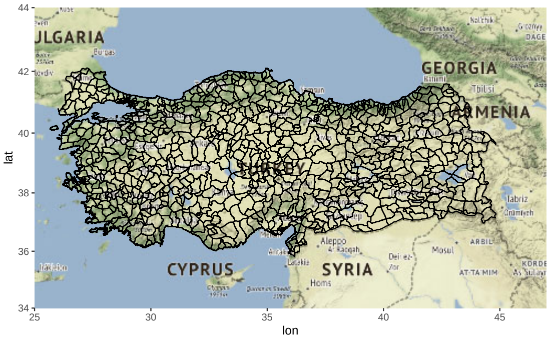

(b) ADM2

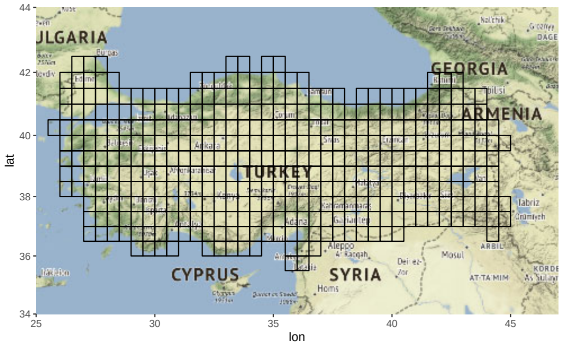

(c) PRIOGrid 0.5

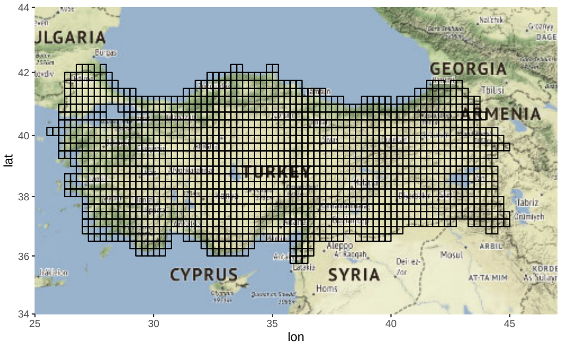

(d) PRIOGrid 0.5

Figure A17: Units of analysis in Turkey.
