# Supplementary material for: A Monte Carlo analysis of false inference in spatial conflict event studies
Source: PLoS One. 2022 Apr 5;17(4):e0266010. doi: 10.1371/journal.pone.0266010 (PMC8982878; doi:10.1371/journal.pone.0266010)
Supplement: Attachment — Submitted filename: review_plos_spatial_simulation.pdf [file pone.0266010.s003.pdf]

Review of A Monte Carlo Analysis of False Inferences in Spatial Conflict Event Studies

In this paper, the authors take on a difficult problem in spatial statistics that appears to significantly affect a large number of studies in political science. While I like the approach that the authors take to address this problem – building a simulation of spatial processes and testing against models – there are issues in the design and analysis of the simulation that need to be addressed before the article can move towards publication.

The main challenge in using a simulation for this type of analysis is having a simulation that adequately captures the DGP of interest. The authors clearly have the substantive expertise necessary to design such a DGP; however, I did not see in the presentation of the DGP a compelling reason why this DGP was chosen and not others.

The linear model part of the DGP seems obvious but is a sensible choice. However, the DGP also includes a censoring mechanism in which we only observe events if a threshold $rnd_{i,t}$ is passed. It was not clear to me why this is included as exactly what this censoring says about the DGP and why it is necessary. Furthermore, this kind of censoring will mean that using a linear model on the data, as the authors later do, will necessarily involve model mis-specification. If the authors have this step in the DGP, then it should also be in the linear model (i.e. use a hurdle model as a first stage). If the intention is to generate counts, then the counts should be drawn from a Poisson process or better yet, to match the estimating equation, the Normal distribution.

The use of military bases is quite clever as a way of inducing spatial autocorrelation. However, other parts of the simulation design were not as clear. The authors do not clearly define how they induce diffusion across cells in the simulation, leaving the reader to guess at what exactly that means and how it affects the simulation.

A major problem with the analysis of the simulation is that the authors do not seem to be employing significance tests correctly with a simulation. A linear model will test whether the coefficient B_1 is different from H_0, i.e. that $B_1 = 0$. However, that should only result in a 5% false significant rate if the coefficient B_1 *is actually zero*. It appears that was only true in one simulation, namely when "complete spatial randomness" applied. For other times, the expected number of non-significant p-values would be quite small if the true value for B_1 were far larger than zero. The authors could possibly adjust for this by using a different H_0 than $B_1 = 0$. They could also consider using coverage, i.e., whether the CI of the linear model includes the true value of B_1 (perhaps that is what the authors in fact did and I am misreading the article).

As such, without this adjustment it can be somewhat difficult to interpret the simulation results as it is not entirely clear what numbers for these parameters we *should* expect. I think a better way to do this would be to focus more on bias than on solely statistical significance. I.e., under what specifications do we get a value for B_1 that is close to the true value? Presumably researchers care not only about detecting an effect but also to what extent conflict events are caused by the count of population. I am also concerned about bias because the estimating equations are different than the simulation; I first would like to see that the researchers can recover a B_1 from a linear model given a simulated dataset. I would encourage the researchers to consider using Gelman and Carlin (2014) work in this area as they recommend four different statistics that collectively cover both inference and bias when evaluating a simulation.

An additional note on the simulation is that it was not entirely clear to me how areal and aggregation-based errors are induced in the data. The use of real-world shapefiles is excellent but the reader needs to understand

exactly what kind of biases are introduced, and how that differs from what the simulation produced and why.

Finally, some general recommendations are to shorten the introduction/background and go into the simulation in more detail in ways that answer some of the questions above. It should be clear to the reader exactly how the data are generated. I also thought the practical advice about spatial event analysis should be moved from SI to the main paper as that is what empirical researchers will primarily want to know from the paper.

References

Gelman, Andrew, and John Carlin. 2014. "Beyond Power Calculations: Assessing Type s (Sign) and Type m (Magnitude) Errors." *Perspectives on Psychological Science* 9 (6): 641–51.
