# Supplementary material for: A Monte Carlo analysis of false inference in spatial conflict event studies
Source: PLoS One. 2022 Apr 5;17(4):e0266010. doi: 10.1371/journal.pone.0266010 (PMC8982878; doi:10.1371/journal.pone.0266010)
Supplement: Attachment — Submitted filename: Event_Simulation_Paper_Revision_Memo.pdf [file pone.0266010.s004.pdf]

REVISION MEMO: PONE-D-21-13937
"A MONTE CARLO ANALYSIS OF FALSE INFERENCE IN SPATIAL CONFLICT EVENT STUDIES"

We would like to thank the editor and the reviewer for their careful reading of our paper. The comments are very helpful. We are particularly grateful for the reviewer's suggestions on how the manuscript may be improved. We concur with all of the points raised, and have incorporated these suggestions into a revised manuscript, which we submit here for your review. To assist you in reviewing the manuscript, we have detailed the specific concerns raised by each party, followed by our response and a list of changes made. We have also provide a new version of the manuscript that highlights all the changes we have implemented in bold text.

General correction

Based on the feedback provided by the editor, the reviewer, and our own reading of the manuscript, we have come to realize that the presentation of the Data Generating Process (DGP) for the simulation was not ideal: both our elaborations in the text and the mathematical definition of the DGP have left the impression that the simulated DGP and the one assumed in the regression analysis were identical. They are not. Instead, we simulate individual conflict events as a string of binary responses of very small simulated locations. These binary events are then aggregated into much larger artificial cells. At the level of these larger cells, OLS regression produces better goodness-of-fit than alternative estimators, because event counts per cell are usually in the tens of hundreds of events. This crucial assumption is now validated in benchmarks. While OLS models are correctly specified, the spatial aggregations in themselves create erroneous inference. Therefore, instead of simulating for the regression analysis, we simulate at the event level and present the problems arising in spatial aggregation and analysis. We elaborate on the details below, but have additionally clarified this approach better throughout the manuscript. Additionally, we have added a new figure to subsection "Design of the conflict event simulator" (see Figure 1).

Reviewer 1

(1) *In this paper, the authors take on a difficult problem in spatial statistics that appears to significantly affect a large number of studies in political science. While I like the approach that the authors take to address this problem – building a simulation of spatial processes and testing against models – there are issues in the design and analysis of the simulation that need to be addressed before the article can move towards publication.*

We thank the reviewer for their overall positive assessment of the manuscript and we have done our best to address the remaining shortcomings. In some instances, we have failed to communicate our approach effectively and we have clarified the corresponding sections in the manuscript.

(2) *The main challenge in using a simulation for this type of analysis is having a simulation that adequately captures the DGP of interest. The authors clearly have the substantive expertise necessary to design such a DGP; however, I did not see in the presentation of the DGP a compelling reason why this DGP was chosen and not others.*

Date: November 11, 2021.

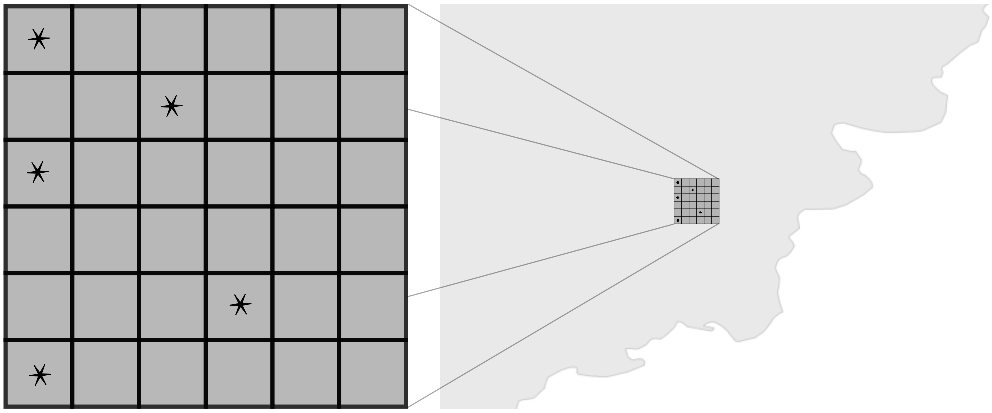

FIGURE 1. **Event representation in SCEG.** At the smallest level, the SCEG simulator represents a lattice of simulated locations orders of magnitude smaller than the areal units used for statistical analysis. In one time-step of the simulation, all simulated cells inside the country potentially generate a conflict event associated with their location.

We fully agree with the reviewer's assessment and have spent a lot of time weighing different mechanisms for simulating event data. We have tried to keep the setup as simple as possible, while maintaining substantively informed mechanisms. In practice, this meant we only focused on one spatial predictor (local population numbers). This variable has been consistently found to be positively correlated with conflict event counts across research designs. We did not include other covariates to eliminate the inference problems stemming from multivariate model specification, rather than Spatial Event Aggregation (SEA) designs themselves.

Similarly, we decided to have simple Bernoulli trials in place to generate conflict events. By running the simulation in multiple steps, repeated trials can generate multiple subsequent conflict events in the same simulated location. This mechanism nicely maps onto the empirical reality of event data collections. To prevent double counting of events reported in separate media outlets, several collections resort to "one-a-day" de-duplication. We also enforce a "one-a-step" limit at the level of simulated locations, but allow for multiple event for the same location over several time steps.

As the reviewer correctly states, the distribution of event counts at the level of simulated locations is therefore not Gaussian (they look Poisson distributed). However, as customary in SEA designs, we do not analyze events at this level. Instead, we spatially aggregate event counts to PG0.5, AMD2, etc. At these higher unit-levels, event counts are generally high and too overdispersed for Poisson models. Event counts at the level of larger spatial units are highly idiosyncratic to geography (country, cell size, and spatial variable). For our set of computational experiments, OLS produces the best fit. In a newly added section to the SI ("Model comparison for the regression analysis"), we document that linear models provide a better fit than Poisson models, for most countries and units in the simulation. The section reads as follows:

We chose linear models to regress event counts on population numbers at the level of PRIO-GRID and admin units. As event counts and variance in the cross-sectional analysis are generally high, we presumed linear models would result in better fits than count-dependent variables models, such as Poisson

models. We validate this assumption below in figure 2 on page 3. We find that linear models usually result in lower AIC scores (i.e. better fit) than Poisson models. For this comparison, we re-estimated both model types as GLMs for experiments 1a and 3a, both of which feature a true population effect.

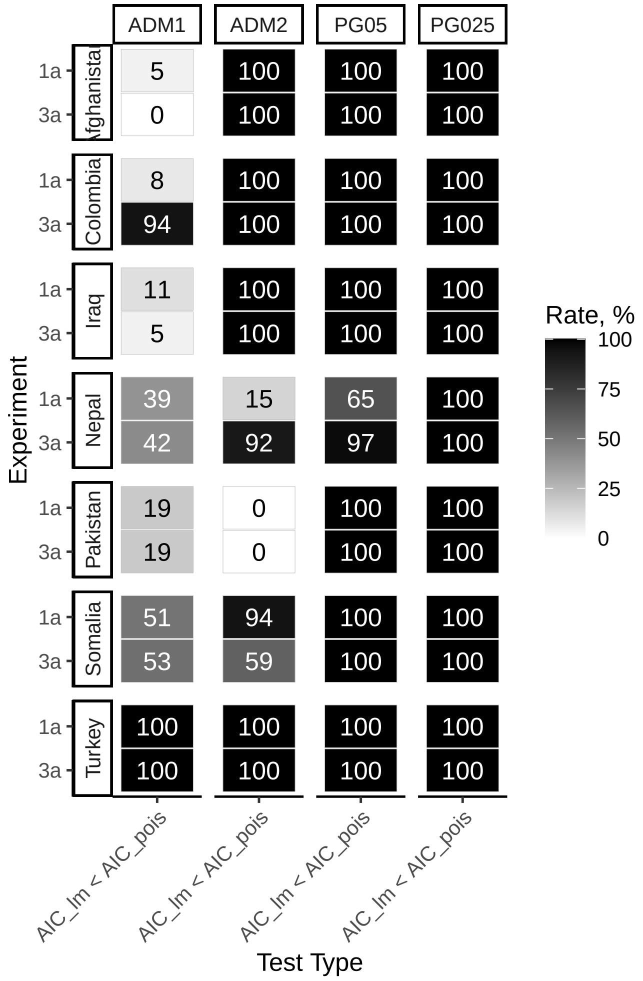

Figure 2. **Comparison between linear models and Poisson models for the regression of event counts on population numbers for PRIO-GRID and administrative units. We find that for most simulated countries and units, linear models perform better in AIC comparisons. This is likely due to the fact that event counts and variance in our cross-sectional analysis tend to be high.**

We now point to this newly added section in an expanded footnote 12 in the main manuscript: **We empirically validate this assumption in Section 4 of the supplementary information.**

Finally, we resort to cross-sectional analysis, again to eliminate problems outside of our methodological scope. We believe that this setup allows us to do justice to the empirical realities of event data collections while keeping the inference as simple and transparent as possible.

We have extended the corresponding discussion in the draft. The section "Data generating process" has been renamed into "Simulated event-generating process" and two new paragraphs convey the overall logic outlined above:

The event-level DGP was designed to be as simple as possible, while still featuring substantively informed mechanisms. In substantive terms, we consider the most basic "civil war": random individuals generate conflict events in a series of time steps. Higher population numbers therefore cause more conflict events when the population effect is employed. We do not include other covariates to eliminate the inference problems stemming from multivariate model specification, rather than SEA designs themselves.

Similarly, we decided to have simple Bernoulli trials in place to generate conflict events. By running the simulation in multiple steps, repeated trials can generate multiple subsequent conflict events in the same simulated location. This mechanism nicely maps onto the empirical reality of event data collections. To prevent double counting of events reported in separate media outlets, several collections resort to "one-a-day" de-duplication. We enforce a "one-a-step" limit at the level of simulated locations, but allow for multiple events for the same location over several time steps. Again, to keep things simple, we omit modeling of temporal dependence for our experiments.

(3) *The linear model part of the DGP seems obvious but is a sensible choice. However, the DGP also includes a censoring mechanism in which we only observe events if a threshold* $rnd_{i,t}$ *is passed. It was not clear to me why this is included as exactly what this censoring says about the DGP and why it is necessary. Furthermore, this kind of censoring will mean that using a linear model on the data, as the authors later do, will necessarily involve model mis-specification. If the authors have this step in the DGP, then it should also be in the linear model (i.e. use a hurdle model as a first stage). If the intention is to generate counts, then the counts should be drawn from a Poisson process or better yet, to match the estimating equation, the Normal distribution.*

This mechanism implements the Bernoulli trials at the level of simulated locations. The reviewer is correct that this can lead to censoring: If the simulation parameters are chosen such that even small population numbers generate a conflict event with near certainty, then locations with higher population numbers will also only produce a single conflict event in any one trial. We mitigate this problem in two ways: first, we chose parameters generally to re-create empirically observed numbers of events. To do this, we factor in the total expected number of events, total population counts, number of simulated locations, and the number of simulated time steps.

We therefore have no use-case where astronomically high event numbers need to be simulated. Second, we generate a series of events over the course of several steps (10 for the current batch of results). The number of trials was chosen to minimize the number of simulated locations producing 10 events in 10 trials, given the event probabilities necessary to recreate the empirically observed N.

The distribution of counts across simulated locations is now shown in a new section in the supplementary information and we include an example figure for reference below. Generally, we see no substantive censoring based on the chosen parameters (see Figure 3). As discussed

above, event counts at the level of larger units are best modelled in a linear regression framework.

We convey this information as follows in a new Section 3 of the supplementary information:

We simulate conflict events in small simulated locations as a series of Bernoulli trials: in several steps (ten in our case), simulated locations get a chance to 'produce' a conflict event. If parameters were chosen incorrectly, this could lead to censoring. If the simulation parameters are chosen such that even small population numbers generate a conflict event with near certainty, then locations with higher population numbers will also only produce a single conflict event. We mitigate this problem in two ways: first, we choose parameters generally to re-create empirically observed numbers of events. To do this, we factor in the total expected number of events, total population counts, number of simulated locations, and the number of simulated time steps.

We therefore have no use case where astronomically high event numbers need to be simulated. Second, we generate a series of events over the course of several steps (10 for the current batch of results). The number of trials was chosen to minimize the number of simulated locations producing 10 events in 10 trials, given the event probabilities necessary to recreate the empirically observed N.

We have double-checked that our parameter choices work. In the figures on page 6 we show that we do not encounter ceiling effects due to censoring at the level of simulated locations for the simulated countries (first run, experiment 1a).

(4) *The use of military bases is quite clever as a way of inducing spatial autocorrelation. However, other parts of the simulation design were not as clear. The authors do not clearly define how they induce diffusion across cells in the simulation, leaving the reader to guess at what exactly that means and how it affects the simulation.*

We agree with the reviewer that the diffusion mechanism has been explained insufficiently. We have expanded the corresponding discussion and it now reads:

To model this effect, we have implemented a mechanism for outcome diffusion in the simulation. Events generated by simulated locations are not recorded locally. Instead, another location within a specific geographic radius is chosen as the attack site. In other words, a locally caused conflict event is not necessarily recorded in the corresponding simulated location. With a certain probability, a simulated location within a predefined radius is selected to record the event (see Figure 4).

Similarly, we have expanded the discussion of error correlation:

A final mechanism implemented in the simulation accounts for latent correlated predictors. As discussed above, this problem can arise from unmodeled processes that are spatially autocorrelated. As suggested in qualitative studies, one underlying mechanism involves the presence of military bases whose locations are not known. Such bases can have an escalating or a deescalating effect on regional conflict intensity: if under attack, they enable military actors to mobilize more resources for fighting. However, military bases can also deter attacks in their vicinity and thereby contribute to areas of low conflict activity.

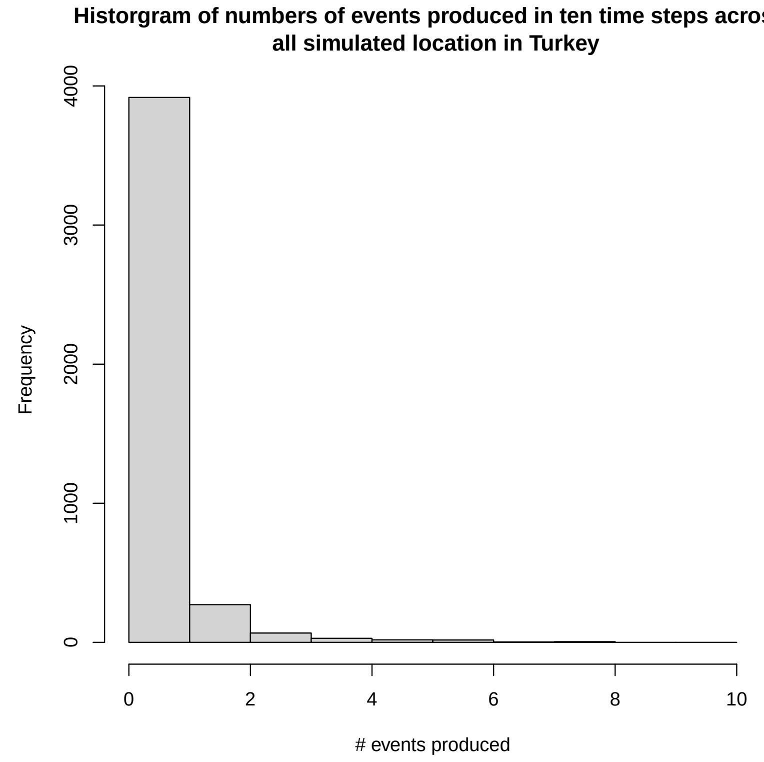

Figure 3. Example histogram of event counts for simulated locations for Turkey. All seven countries are analyzed like this in section 3 of the supplementary information.

The net effect of the presence of bases is likely to affect several spatial units, especially when units are small (see Figure 4).

A new figure 4 has been added to convey the workings of these simulated mechanisms visually.

(5) *A major problem with the analysis of the simulation is that the authors do not seem to be employing significance tests correctly with a simulation. A linear model will test whether the coefficient B_1 is different from H_0, i.e. that $B_1 = 0$. However, that should only result in a 5% false significant rate if the coefficient B_1* ***is actually zero****. It appears that was only true in one simulation, namely when "complete spatial randomness" applied. For other times, the expected number of non-significant p-values would be quite small if the true value for B_1 were far larger than zero. The authors could possibly adjust for this by using a different H_0 than $B_1 = 0$. They could also consider using coverage, i.e., whether the CI of the linear model includes the true value of B_1 (perhaps that is what the authors in fact did and I am misreading the article). As such, without this adjustment it can be somewhat difficult to interpret the simulation results as it is not entirely clear what numbers for these parameters we* ***should*** *expect.*

The reviewer eloquently summarized the logic of our significance tests. Upon re-reading the manuscript, we have realized that we have not conveyed this logic prominently enough in the original manuscript: the "Computational Experiments" section says that we also conduct experiments where population had no effect on conflict intensity, but we fail to state that each experimental condition is tested with and without a population effect. Table

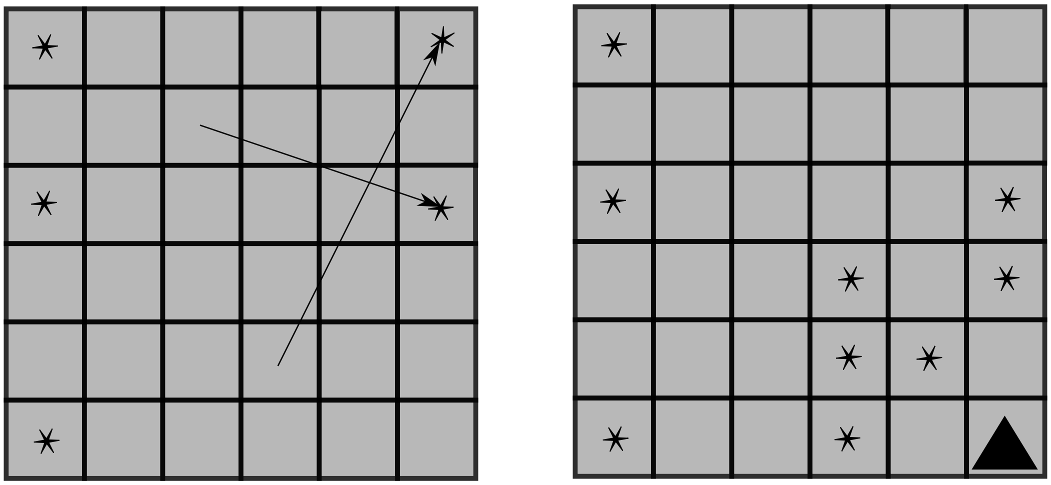

Figure 4. **Diffusion and error correlation in SCEG.** On the left, the diffusion mechanism is illustrated: with a 0.5 probability, locally caused conflict events will experience "outcome diffusion" and relocate to a random simulated location within a predefined radius. On the right, the presence of a military base indicated by the triangle in the lower right corner causes more conflict events in its vicinity.

2 conveys the information, but the text and the table are more explicit in the new version of the manuscript:

Importantly, we also ran corresponding scenarios where local population counts had no effect: each experiment summarized in table 2 was conducted with population causing conflict events and in a control condition where population has no effect. In these latter scenarios, conflict events were only the product of complete spatial randomness caused by α_0 in equations 1 and 4, as well as diffusion and error correlation. The "Effect" and the "Summary" columns in table 2 communicate these important distinctions that are crucial for subsequent statistical analysis.

(6) *I think a better way to do this would be to focus more on bias than on solely statistical significance. I.e., under what specifications do we get a value for B_1 that is close to the true value? Presumably researchers care not only about detecting an effect but also to what extent conflict events are caused by the count of population. I am also concerned about bias because the estimating equations are different than the simulation; I first would like to see that the researchers can recover a B_1 from a linear model given a simulated dataset. I would encourage the researchers to consider using Gelman and Carlin (2014) work in this area as they recommend four different statistics that collectively cover both inference and bias when evaluating a simulation.*

In the preparation of the original manuscript, we did have internal discussions along very similar lines! We considered both the approach laid out the reviewer, as well as a more general analysis of statistical power, i.e. the question whether a signal can be detected, if a true causal effect is present.

We decided against prioritizing these approaches for three different reasons. First and most importantly, our main concern with this research design is that it easily produces false positives: effects are claimed to exist, even though they do not. In our view, this

problem justifies a strong emphasis on the frequencies of falsely rejected null hypotheses in the manuscript, and we hereby follow standard statistical practices where low α rather than low β-error probability is prioritized. In the "Results and Discussion" section, we compare the observed rates of false positive results to the assumed probabilities of such results under a 5% α-error probability, for cases where no population effect is present. We do discuss high rates of false negative effects when the population effect is present, but without reference to explicit calculations of power, effect size, or β-error probability.

Beyond this general focus, we also have technical reasons to abstain from such explicit calculations. Given that we analyze causal effects in four different artificial spatial cell types for seven different countries, we would have to repeat these calculations for all cells types and countries as a function of the available number of observations and the modeled effect size. The effects specified at the level of simulated locations can be kept constant, but SEA designs generate inference for much larger spatial units. Depending on the size and geometry of the units, their 'causal effect' can drastically vary, even if the population-level event probabilities are kept constant.

Given that the events are simulated in *NetLogo*, while the statistical analysis is performed in *R*, these calculations would require serious technical investment. In light of the secondary priority that we assign to this, we have abstained from this investment.

Finally, we do show that statistical power is affected by spatial aggregations: false negative inference in Iraq and Somalia, for example, is heavily affected by the choice of administrative units (ADM-1 vs. ADM-2). Even though we do not calculate the exact number of permissible false negative results as a function of number of observations and effect size at these levels of aggregation, we do observe large differences between these chosen units. Admittedly, we can discuss this problem only qualitatively with our current design. However, the large differences between false negative rates as a function of unit choice reveals that statistical power is unreliable in SEA designs.

In response to the reviewer's comment, we have added a disclaimer to the "Statistical analysis" section:

These false positive results are the main focus of our computational experiments, because they are most damaging to the overarching research program. However, we also discuss the problem of false negative results in SEA designs. A direct comparison of expected probabilities and observed frequencies would require statistical power calculations for each country and type of unit. Instead of providing those, we simply discuss variation in the rates of false negative results as a function of unit size and simulated country.

Also, we have strengthened the associated discussion in the "Summary and Discussion" section:

For false positive results, this problem is immediately apparent when comparing the assumed 0.05 probability of such errors with their much higher observed frequency. For false negative results, more involved power calculations would be required to make an analogous comparison. However, we observe strong variation in the observed frequency of such errors under identical experimental conditions, simply as a function of different spatial units.

(7) *An additional note on the simulation is that it was not entirely clear to me how areal and aggregation-based errors are induced in the data. The use of real-world shapefiles is excellent but the reader needs to understand exactly what kind of biases are introduced, and how that differs from what the simulation produced and why.*

To demonstrate the problems introduced by spatial aggregation, we simulate individual conflict events in large numbers. We then aggregate them into spatial units, thereby emulating the workflow in SEA designs.

In doing so, we uncover problems resulting simply due to spatial aggregation into artificial cells, and problems that result from outcome diffusion and error correlation.

This overall logic was not presented ideally in the initial version of the draft and we hope that we have clarified the research design with the addition of the figures and the rewording of the main text. In particular, we have strengthened the distinction between event-level simulation and unit-level analysis as discussed above. We have also reworded the discussion around the simulated DGP which necessarily differs from the statistical notion of DGP at the unit level. To this end, we have renamed the subsection "Data generating process" into "simulated event-generating" process and we now refer to "event-level simulations" throughout the manuscript.

(8) *Finally, some general recommendations are to shorten the introduction/background and go into the simulation in more detail in ways that answer some of the questions above. It should be clear to the reader exactly how the data are generated. I also thought the practical advice about spatial event analysis should be moved from SI to the main paper as that is what empirical researchers will primarily want to know from the paper.*

We have restructured the manuscript accordingly, although we have struggled with the streamlining of the introduction. We believe that contemporary event data collections should be mentioned and that the overall goals of our study need to be communicated. An obvious subsection to shorten would be "origins and prominence of SEA designs", but here we also introduce the PRIO-GRID and GADM data collections. Substantive engagement with previous studies has already been relegated to the Supplementary Information. This leaves only Table 1 as a suitable element to cut from the main text, but it is referenced later in the text several times. We are open to moving this table to the Supplementary Information in a future iteration.

We have done our best to clarify the internal workings of the simulation as outlined above.

The practical advice section from the SI has been worked into the main manuscript as a new subsection named "Practical implications for applied SEA studies" under "Results and discussion".
