# Supplementary material for: A Monte Carlo analysis of false inference in spatial conflict event studies
Source: PLoS One. 2022 Apr 5;17(4):e0266010. doi: 10.1371/journal.pone.0266010 (PMC8982878; doi:10.1371/journal.pone.0266010)
Supplement: Attachment — Submitted filename: review_plos_spatial_simulation_v2.pdf [file pone.0266010.s005.pdf]

Review of A Monte Carlo Analysis of False Inferences in Spatial Conflict Event Studies, Round II

The authors may consider the following points for clarification:

1. The abstract and introduction should contain the main takeaways of the paper, which appear to be that 1) the most efficient and robust estimator is a spatial lag model with an areal control and 2) that problems of spatial diffusion can also be effectively mitigated with either using larger aggregation sizes or employing over-time inference with district-level fixed effects.
2. In Eq. (1), presumably there is a logit link function (or equivalent); it should be included in the formula as in $g(\alpha_0 + \alpha_1 x_i))$.
3. Please use more informative labels than just Experiment 1a or 1b. For example, say Experiment 1a (no diffusion, areal confounding) so that the reader can follow along more easily without having to refer back to the table constantly.
4. The text should explicitly state that the simulation is evaluated in terms of inference on α_1. To facilitate that, I would urge the authors to revise Eqs. 5-7 to use hat notation, as in $Y = \hat{\alpha_0} + \hat{\alpha_1} x_i$ to make it clear that these are estimated parameters of the DGP. (Note too that the subscript i on x was dropped in 5-7 but presumably should be included).
5. The FPs and FNs should be defined notationally in the text and also in the figure legends, as in FP = $Pr(\hat{\alpha_1} > 0|\alpha_1 = 0)$ and FN = $Pr(\hat{\alpha_1} = 0|\alpha_1 \neq 0)$.
6. The discussion of FNs is somewhat confusing as it's not as clear what the true/acceptable FN rate should be (perhaps we use the standard convention of 80% power?). Estimators can be conservative but people don't always think that's a problem from an inference perspective. I might suggest the authors compile an ROC plot showing FN/FP trade-offs across varying sample sizes or some other criterion. This would be additional work but would be more helpful than simply evaluating a single number for FNs. For example, an FN of 100% will mechanically mean no FPs because the estimator never identifies an effect.
