# Supplementary material for: A Monte Carlo analysis of false inference in spatial conflict event studies
Source: PLoS One. 2022 Apr 5;17(4):e0266010. doi: 10.1371/journal.pone.0266010 (PMC8982878; doi:10.1371/journal.pone.0266010)
Supplement: Attachment — Submitted filename: revision_memo2.pdf [file pone.0266010.s006.pdf]

REVISION MEMO: PONE-D-21-13937R1 "A MONTE CARLO ANALYSIS OF FALSE INFERENCE IN SPATIAL CONFLICT EVENT STUDIES", ROUND II REVISIONS

We would like to thank the editor and the reviewer for their careful reading of our paper. We are particularly grateful for the reviewer's suggestions on how the manuscript may be improved. Even in round 2, the reviewer's comments speak to the most crucial and least intuitive aspects of the study and have greatly improved the overall clarity of our approach.

General corrections

We have corrected several typos throughout the manuscript and have done our very best to clarify the simulation approach. We believe that these clarifications strongly improve the transparency of the research design, especially in combination with the figures and explanations provided for the initial revision.

Reviewer 1

(1) *The abstract and introduction should contain the main takeaways of the paper, which appear to be that 1) the most efficient and robust estimator is a spatial lag model with an areal control and 2) that problems of spatial diffusion can also be effectively mitigated with either using larger aggregation sizes or employing over-time inference with district-level fixed effects.*

We fully agree and have added the following paragraph to the abstract:

Controlling for the geographic area of spatial units eliminates an important source of spurious correlation. In time-series analyses, the same result can be achieved with unit-level fixed effects. Under outcome diffusion, spatial lag models with area controls produce most reliable inference. When those are computationally intractable, geographically larger aggregations lead to similar improvements. Generally, all analyses should be performed at two separate levels of geographic aggregation.

(2) *In Eq. (1), presumably there is a logit link function (or equivalent); it should be included in the formula as in* $g(\alpha_0 + \alpha_1 x_i)$.

We concede that a link function in Equation (1) would be required if we attempted to efficiently estimate the true α-parameters in the subsequent statistical analysis. However, this is generally not done in SEA designs, because the data-generating process is presumed to operate on smaller scales than the data analysis. Local conditions presumably cause conflict. The presence and direction of an effect is then established in sign and significance of larger-scale correlations, for instance at the ADM2-level.

We have therefore implemented a causal effect without the need for a link function: we simply generate a certain percentage of events as a function of local population. This is a true causal effect, but the associated multiplier, α_1, is not directly recovered in the subsequent regressions, because they operate at larger spatial scales. Still, the presence of

Date: March 5, 2022.

a true effect is visible in sign and significance of the β_1-parameters in the regressions. We have strengthened the write-up of the paper to convey this important point to readers:

In "Simulated event-generating process": **More importantly, we can calibrate α-parameters to implement causal effects. As the total population per country, the number of simulated locations, the number of simulated steps, and the empirical number of conflict events are all known, we can ensure that population counts in each cell cause large fractions of all simulated events by simply adjusting α_1. For instance, we can divide the empirical number of conflict events by 2, when attributing 50% of events to population in the computational experiments. We then divide that number by the total population of the country and the number of simulation steps. The resulting fraction is the per-capita, per-step probability of generating a conflict event.**

Similarly, we can control unexplained variance in event counts by choosing α_0 accordingly. This calibration also eliminates the need for a link function in Equation 1 and statistical estimation in identifying parameters in the simulated event-generating process. Once those parameters are set, a continuous random number is drawn from a uniform distribution starting at zero and ending at one for each simulated cell i within the borders of a simulated country, for each step t:

We repeat this point in "computational experiments": **For the results presented below, we calculate population totals for each country, as well as the number of simulated locations within country boundaries. This allows us to choose α-parameters in Equation 1 to approximately replicate empirical event counts. We recreate the empirical counts in the following way: 50% of total simulated events arise from the baseline probability of any simulated location producing a conflict event in any time step. Another 50% are generated based on the population effect. This means that event counts differ depending on whether or not the population effect is simulated, but effect sizes are constant across experiments.**

(3) *Please use more informative labels than just Experiment 1a or 1b. For example, say Experiment 1a (no diffusion, areal confounding) so that the reader can follow along more easily without having to refer back to the table constantly.*

This is a very good suggestions and we have added corresponding summaries throughout the paper. Most importantly, we have added them to all figure captions, where they are critically important for interpretation of results, but we have also added half-sentence summaries to all experiments mentioned in the text.

(4) *The text should explicitly state that the simulation is evaluated in terms of inference on α_1. To facilitate that, I would urge the authors to revise Eqs. 5-7 to use hat notation, as in $Y = \hat{\alpha_0} + \hat{\alpha_1} x_i$ to make it clear that these are estimated parameters of the DGP. (Note too that the subscript i on x was dropped in 5-7 but presumably should be included).*

The reviewer's suggestion would make for a very elegant formal presentation of the analysis. Unfortunately, the estimation is not quite as straight forward. The α-parameters in the simulation and the β-parameters in the estimation are obviously related, but the latter is not directly an estimate of the former. This has to do with the spatial aggregation in SEA designs. SEA designs presume that local conditions cause local conflict events. In the

simulations, we do justice to this assumption by using a fine-grained lattice of simulated locations (depicted in Figure 4). These locations generate conflict events in a series of time-steps. However, resulting event counts are not directly regressed on the local variables in each simulated location. Instead, both event counts and covariate values are spatially aggregated into larger units of analysis, such as PG05 or ADM2. The β-parameters in equations 5-7 are estimated for these much larger units and only evaluated in terms of sign and significance.

This has been previously mentioned in passing on page 1, but we now reiterate this important point in the "materials and methods" section: **We use those datasets to benchmark SEA designs: we aggregate events into artificial cells of varying types and sizes, and then estimate the effect of population figures on event intensity. We only rely on sign and significance to identify causal effects, just as the reviewed SEA studies. All experiments were conducted with and without a true causal effect, which allows us to establish rates of correct and incorrect inference across multiple simulation runs.**

We had several discussions of whether we can formally express the β-parameters as a function of $\sum \alpha_1 x$ for the simulated locations contained in geographic units. However, what is being summed up comes down to irregularly shaped geographic units. This, in essence, is the Modifiable Areal Unit Problem. We believe it is better to convey this visually and linguistically in the text, rather than formally. However, we remain very open to suggestions on how this can be conveyed better. The reviewer is 100% correct in demanding effective communication of this crucial aspect of the study.

In empirical or simulated SEA designs, α and β are very different variables: α is derived from the share of total conflict events we attribute to local population in the simulations. β expresses the correlation between unit-level counts of population and conflict events.

We have expanded the discussion on this in the "statistical analysis" section:

In these models, the β_1 parameter captures the population effect expressed in α_1 in equation 1. However, β_1 is not directly an estimate of α_1, which is derived from the share of total conflict events we attribute to local population in the simulated locations. Instead, β_1 expresses the correlation between counts of population and conflict events at the level of much larger geographic units, such as PG05 or ADM2.

(5) *The FPs and FNs should be defined notationally in the text and also in the figure legends, as in $FP = Pr(\hat{\alpha_1} > 0|\alpha_1 = 0)$ and $FN = Pr(\hat{\alpha_1} = 0|\alpha_1 \neq 0)$.*

This is an excellent suggestion. The previous discussion of FP and FN was needlessly ambiguous. We have followed the reviewer's advice and added the following paragraph to section "statistical analysis":

Across models, the discussion of false inference is focused on the α_1 in the simulated data-generating process (see Equation 1). The presence and direction of this local causal effect is gauged by the sign and significance of larger-scale correlations at the level of grid-cells or administrative units, i.e. estimates for β_1 in Equations 5-7.

We define false positive results (FP) as simulation runs where no casual effect is present, but we see a positive and statistically significant estimate: $FP : \hat{\beta}_1 > 0|\alpha_1 = 0$. **Analogously, false negatives (FN) are defined as** $FN : \hat{\beta}_1 = 0|\alpha_1 > 0$

This notation deviates slightly from the one suggested by the reviewer, because we believe that retaining the α and β distinction is advantageous. But we fully agree that having a formal definition for FN and FP strengthens the paper.

(6) *The discussion of FNs is somewhat confusing as it's not as clear what the true/acceptable FN rate should be (perhaps we use the standard convention of 80% power?). Estimators can be conservative but people don't always think that's a problem from an inference perspective. I might suggest the authors compile an ROC plot showing FN/FP trade-offs across varying sample sizes or some other criterion. This would be additional work but would be more helpful than simply evaluating a single number for FNs. For example, an FN of 100% will mechanically mean no FPs because the estimator never identifies an effect.*

We see the merit of the reviewer's suggestion, but we have struggled with implementation. An ROC curve works very well with predicted probabilities for a binary outcomes, but we code FN and FP as a function of sign and significance in linear regression of event counts. The effects of this design choice are counter-intuitive: the FN results refer to a version of the experiment where the true causal effect is present, i.e. $\alpha_1 > 0$. In that case, a positive and statistically significant effect should be found for $\hat{\beta}_1$ in a large number of experiments. The exact number of experiments that should lead to significant estimates would require power calculations involving the number of PG or ADM-units under investigation, which varies between experiments and countries.

The FP results refer to a version of the experiment where $\alpha_1 = 0$, as correctly pointed out by the reviewer in the previous point. Here the permissible number of false results under a customary p of 0.05 is much easier to establish, because we can directly compare the observed frequency of FP with their assumed 0.05 probability.

However, FN and FP do not rely on the same data. There are 100 runs dedicated to testing FN and 100 different runs for detecting FP for each experiment. As a consequence, we see FP and FN sum up to more than 100%. In fact, Somalia at the ADM2 resolution in Figure 6 shows 100% FP and 100% FN.

Even if we decided to change the p-values to emulate varying probability cutoffs in a ROC curve, this problem would prevent us from drawing a ROC curve. Additionally, the reviewed SEA designs all rely on p-values of 0.05.

Again, the reviewer has touched on a very important and rather counter-intuitive aspect of our data analysis and we now convey this effect more clearly in a newly added paragraph in the "statistical analysis" section:

One counter-intuitive consequence of our analysis of false inference is that false positives and false negatives can sum to more than 100%: false negatives are established in model runs where $\alpha_1 > 0$ **and we would expect to find an effect. False positives are researched for the same units of analysis and countries, but with** $\alpha_1 = 0$ **in a separate set of runs. We find high rates of both types of errors for certain units and countries.**
